# Progress in peptide and protein therapeutics: Challenges and strategies

**DOI:** 10.1016/j.apsb.2025.10.026

**Published:** 2025-10-25

**Authors:** Mengyang Liu, Darren Svirskis, Thomas Proft, Jacelyn Loh, Naibo Yin, Hao Li, Danhui Li, Yongzhi Zhou, Shuo Chen, Lizhuo Song, Guanyu Chen, Wei-Yue Lu, Zhiwen Zhang, Zhou Zhou, Lian Li, Yuan Huang, Craig Bunt, Guiju Sun, Paul W.R. Harris, Margaret A. Brimble, Jingyuan Wen

**Affiliations:** a School of Pharmacy, Faculty of Medical and Health Sciences, The University of Auckland, Auckland 1023, New Zealand; b Department of Molecular Medicine and Pathology, Faculty of Medical and Health Sciences, The University of Auckland, Auckland 1023, New Zealand; c Maurice Wilkins Centre for Molecular Biodiscovery, The University of Auckland, Auckland 1023, New Zealand; d Gongli Hospital of Shanghai Pudong New Area, Shanghai 200135, China; e School of Pharmaceutical Sciences (Shenzhen), Shenzhen Campus of Sun Yat-sen University, Shenzhen 518063, China; f School of Pharmacy, Fudan University, Shanghai 201203, China; g Key Laboratory of Drug Targeting and Drug Delivery, West China School of Pharmacy, Sichuan University, Chengdu 610041, China; h The Department of Food Science, University of Otago, Dunedin 9016, New Zealand; i Department of Nutrition and Food Hygiene, School of Public Health, Southeast University, Nanjing 210009, China; j School of Biological Science, Faculty of Sciences, University of Auckland, Auckland 1010, New Zealand; k School of Chemical Science, Faculty of Sciences, University of Auckland, Auckland 1010, New Zealand

**Keywords:** Peptides and proteins, Skin barriers, Gastrointestinal barriers, Blood‒brain barriers, Penetration enhancers, Enzymatic inhibitors, Chemical modification, Nanoparticulate drug delivery systems

## Abstract

Peptide- and protein-based therapeutics offer realized and potential benefits to health, due to their potent bioactivity, high specificity, and favorable safety characteristics. However, their widespread clinical application is constrained by inherent limitations, including rapid enzymatic degradation, poor membrane permeability, and a reliance on parenteral administration, which reduces patient adherence. To overcome these challenges, extensive research has explored non-invasive delivery strategies, including topical, transdermal, and oral formulations. Despite promising advances in these delivery strategies, they are yet to overcome substantial biological and physicochemical barriers in peptide and protein therapeutics, such as enzymatic degradation in the gastrointestinal tract, limited epithelial transport, and inherently low systemic bioavailability. This review provides a comprehensive and up-to-date analysis of the structural and physiological barriers influencing peptide and protein bioavailability and therapeutic efficacy. It critically examines key challenges associated with various administration routes, including topical, transdermal, oral (including delivery targeting the brain), and others. Furthermore, it explores innovative strategies to enhance peptide and protein stability and bioavailability, including chemical modifications, enzyme inhibitors, penetration enhancers, physical delivery technologies, and advanced nanoparticulate formulations. Additionally, emerging trends in formulation optimization, regulatory considerations, and translational pathways for clinical implementation are discussed. By addressing these critical challenges and highlighting recent advances, this review serves as a roadmap for the development of next-generation peptide and protein therapeutics with improved stability and efficacy, and enhanced patient adherence, which is needed to fully realize the true potential of this class of therapeutics.

## Graphical abstract

This article offers an in-depth analysis of the key challenges associated with peptide drug delivery, including physical, enzymatic, and membrane-related barriers that limit their clinical effectiveness.

**Figure:**
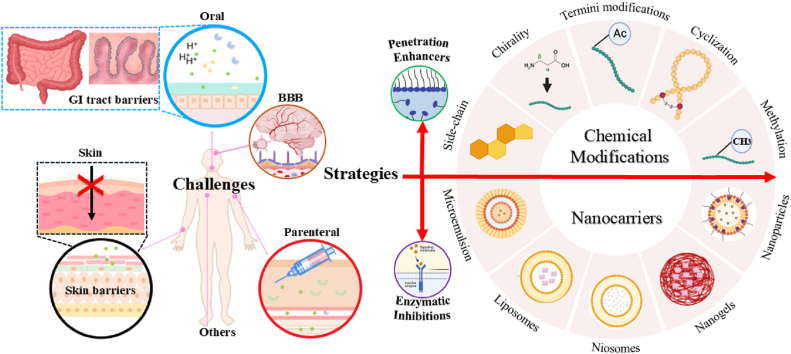

## 1 Introduction

Peptides are short chains of amino acid residues, typically consisting of fewer than 50 amino acids, linked together by peptide bonds^1^. Proteins, on the other hand, are larger biomolecules composed of one or more polypeptide chains, generally exceeding 50 amino acids in length^2^. Compared to small-molecule drugs, peptides and proteins exhibit high specificity in their biological activity, resulting in fewer off-target effects and reduced toxicity. This has led to their increasing application in pharmaceuticals, functional foods, and cosmetics over the past decade. As of 2025, the global peptide and protein therapeutics market continues to expand, with over 80 peptide-based drugs currently approved for clinical use, contributing around $50 billion USD in pharmaceutical revenues^3^. Additionally, more than 150 peptide candidates are in clinical trials, with another 600–700 in preclinical development^4^. The market for peptide and protein-based drugs is expected to expand at a compound annual growth rate (CAGR) of 9% to 10%, exceeding $100 billion USD by 2030^5^. This surge is driven by advancements in drug delivery technologies, bioengineering, and recombinant protein production, making peptides and proteins key players in the future of biopharmaceuticals. In recent years, major pharmaceutical companies have filed more patent applications for biopharmaceuticals than for small-molecule drugs, with this trend continuing to grow. This shift is reflected in global sales data; in recent years, peptide- and protein-based therapeutics, especially monoclonal antibodies, have continued to dominate the pharmaceutical market. For instance, by 2024, drugs such as adalimumab (Humira®, AbbVie) and infliximab (Remicade®, Johnson & Johnson and Merck & Co.) remain among the top-selling biologics worldwide^6^. The global market for monoclonal antibodies alone was valued at over US$200 billion in 2024, and projections reach over US$400 billion by 2029, reflecting sustained growth driven by increasing demand for targeted therapies^6^. These data highlight the expanding clinical and commercial importance of peptide- and protein-based drugs. In fact, seven out of the ten best-selling pharmaceuticals are based on amino acid sequences^6^. The growing preference for larger biopharmaceuticals over traditional small-molecule drugs introduces unique challenges for formulation scientists. Therapeutic peptides and proteins are increasingly being integrated into clinical treatment strategies, owing to their high specificity and ability to target disease pathways with precision^7^. The main challenge in commercializing peptide and protein-based drugs stems from their limited stability, due to both physical and biochemical barriers encountered during oral or transdermal administration^8^. As a result, invasive parenteral routes remain the most viable means of delivery. However, there is no systemic analysis or overview of the different routes of administration for peptide/protein therapeutics. Therefore, this article will highlight recent advancements in the development and application of peptide and protein therapeutics. In particular, the comprehensive challenges in delivering peptides and proteins through different administration routes and the advanced strategies to overcome these challenges are presented and discussed in this review.

## 2 Challenges in peptide and protein delivery

### 2.1 Intrinsic challenges of peptide and protein

Peptides and proteins face considerable hurdles in their development as therapeutic agents, primarily because of inherent physical and chemical instabilities. Key challenges include rapid clearance from the bloodstream, vulnerability to enzymatic degradation, potential immunogenic responses, and loss of therapeutic efficacy resulting from structural conformational changes^9^. The instability of peptides and proteins can result from multiple degradation pathways, including oxidation, deamidation, conformational unfolding, aggregation, and denaturation at interfaces^10^, ^11^, ^12^. Additionally, peptides and proteins are characterized by intricate secondary and tertiary structures, which are essential for maintaining their biological activity and are particularly vulnerable to disruption. Even subtle perturbations to their native structures can lead to a loss of bioactivity, complicating their therapeutic potential^13^. Therefore, the main intrinsic challenges of peptides and proteins are their physicochemical barriers in terms of molecular weight, structure, solubility, stability, immunogenicity, and so on.

#### 2.1.1 Molecular size and weight

Peptides and proteins possess significantly larger molecular sizes compared to conventional small-molecule drugs (<500 Da), which greatly limits their ability to cross biological membranes *via* passive diffusion^14^. Their high molecular weight, bulky structure, and hydrophilicity hinder absorption through non-invasive routes such as oral, nasal, and transdermal pathways. In general, passive transcellular diffusion is most favorable for compounds under 500 Da with high lipophilicity and balanced hydrophilic–lipophilic properties (log*P* between 1 and 3)^15^. Therefore, the inherent size and polarity of peptides and proteins remain major barriers in designing effective non-invasive delivery systems.

#### 2.1.2 Structural complexity and conformational sensitivity

Proteins exhibit complex secondary, tertiary, and often quaternary structures that are essential for their biological function^16^. These higher-order conformations are inherently sensitive to physicochemical stressors such as pH variation, thermal fluctuations, shear forces, and enzymatic degradation^17^. Exposure to such conditions can result in denaturation, aggregation, or misfolding, which not only reduces therapeutic efficacy but may also provoke undesired immunogenic responses^18^. Peptides, while structurally simpler, remain vulnerable to chemical degradation pathways including deamidation, oxidation, and hydrolysis, particularly at asparagine, methionine, or cysteine residues^19^. To maintain therapeutic activity and safety, peptide and protein formulations must preserve conformational integrity across manufacturing, storage, and delivery processes.

#### 2.1.3 Hydrophilicity and solubility

Peptides and proteins are highly hydrophilic macromolecules, which severely limit their membrane permeability and ability to cross epithelial barriers *via* passive diffusion^20^. Their solubility is governed by factors such as amino acid composition, net charge, isoelectric point (pI), and the presence of hydrophobic or amphiphilic domains^20^. Proteins are particularly prone to aggregation and precipitation when the formulation pH approaches their pI, which is commonly between pH 4 and 8, and where the net charge is minimized^21^. To optimize solubility, formulation pH is typically adjusted at least 1 to 2 units close to the pI, thereby increasing repulsive electrostatic interactions and reducing aggregation risk^21^.

#### 2.1.4 Stability

Peptide and protein therapeutics face substantial stability challenges, particularly within biological environments. They are highly susceptible to enzymatic degradation by proteases such as pepsin (active in the stomach), trypsin, and chymotrypsin (active in the intestine and systemic circulation), which rapidly cleave peptide bonds and inactivate the molecule^22^. Additionally, peptides and proteins with molecular weights typically below 60 kDa are subject to rapid renal clearance, contributing to short plasma half-lives and often in the range of min to a few hours^23^. For example, unmodified insulin has a half-life of less than 5 min^23^. These rapid clearance and degradation processes significantly limit therapeutic exposure and necessitate frequent or high-dose administration, which can negatively impact patient adherence and increase treatment burden.

#### 2.1.5 Immunogenicity

Although therapeutic peptides and proteins are often derived from or structurally similar to endogenous human biomolecules, they can still elicit undesired immune responses, especially when their structure or presentation is altered during production, formulation, or delivery^24^. Modifications such as non-native glycosylation patterns, oxidation of methionine or tryptophan residues, deamidation, or protein aggregation can introduce neoepitopes or expose normally hidden regions of the molecule, triggering recognition by the immune system^24^^,^^25^. Aggregated proteins, in particular, have a high potential to activate antigen-presenting cells and initiate adaptive immune responses. Immunogenicity may lead to the production of neutralizing antibodies, which can reduce or abolish the therapeutic effect, and in some cases result in hypersensitivity reactions or autoimmunity^24^^,^^26^. Risk factors include the degree of sequence homology to endogenous proteins, route of administration (with subcutaneous and intramuscular routes typically more immunogenic than intravenous), dose frequency, and patient-specific factors such as genetic background and immune status^25^^,^^26^.

External challenges, including formulation stability, handling requirements, cold-chain storage, and transport logistics, pose significant hurdles to the successful deployment of peptide- and protein-based therapeutics^27^. These issues are further compounded by the distinct physicochemical sensitivities of such biopharmaceuticals, necessitating route-specific considerations. Various routes, such as topical/transdermal, oral, and parenteral administration, each present unique challenges in terms of peptide/protein stability and bioavailability. Examples of specific peptides/proteins and their challenges across different routes of administration are summarized and provided in Table 1 28, 29, 30, 31, 32, 33, 34, 35, 36, 37, 38, 39, 40, 41, 42, 43, 44, 45, 46, 47. These challenges are further compounded by the complexity of ensuring effective delivery without compromising the peptide/protein's therapeutic efficacy. For instance, peptides and proteins administered orally must contend with harsh gastrointestinal conditions, while those administered topically or transdermally must overcome skin barriers to achieve systemic absorption. To address these challenges, an extensive and deep understanding of the physical, chemical, pharmaceutical, and biological factors influencing peptide/protein stability and absorption is essential. This knowledge is essential for the rational design of therapeutic peptides and proteins. In this article, we examine the key obstacles and limitations involved in delivering peptide/protein drugs *via* various routes, providing insights into strategies for overcoming these obstacles. The discussion highlights the importance of tailored approaches for each route of administration to ensure the successful application of peptide/protein therapeutics.

**Table 1:** Examples of peptides/proteins and their administration challenges across various routes^28^, ^29^, ^30^, ^31^, ^32^, ^33^, ^34^, ^35^, ^36^, ^37^, ^38^, ^39^, ^40^, ^41^, ^42^, ^43^, ^44^, ^45^, ^46^, ^47^.

Route	Example	Formulation	Clinical application	Delivery strategy	Challenges in delivery	Ref.
Skin	Insulin	Microneedles, hydrogel, liposomes	Diabetes management	Microneedle arrays, iontophoresis, ultrasound	Low permeability of large molecules, enzymatic degradation, and stability in skin layers	28, 29, 30
	Calcitonin	Liposomes, patches	Osteoporosis treatment	Iontophoresis, encapsulation	Limited absorption through the stratum corneum, stability during formulation	31,32
	Collagen	Hydrogel	Wound healing, anti-aging	Passive diffusion, crosslinking hydrogel networks	Degradation by skin proteases, inefficient transdermal delivery	33
	Leuprolide acetate	Polymeric microspheres	Prostate cancer, endometriosis	Controlled-release microneedles	Pain with larger dosages, difficulty maintaining sustained release in microenvironments	34
	Argireline	Molecular modification	Reduce wrinkle	Chemical modification to increase lipophilicity	Low permeation of argireline through skin due to its large molecular weight and hydrophilicity	41
Oral	Insulin	Penetration enhancer salcaprozate sodium (SNAC)	Diabetes	Permeation enhancer to open the tight junctions	Intestinal epithelium barrier, high molecular weight of insulin	35, 36, 37
	GLP-1 analogs	Lipid nanoparticles	Type 2 diabetes, obesity	Lipase protection, polymer nanoparticle encapsulation	Low intestinal permeability, susceptibility to first-pass metabolism	38
	Linaclotide	Capsules	Chronic constipation	Enteric coating protection	Swallow issues and low bioavailability	47
	Exendin-4	pH-triggered hydrogel	Anti-diabetes	Large pore size and surface area of nanoparticles enhance drug loading, and protection of nanoparticles from the acidic environment of the stomach	Acidic environment and pepsin-mediated degradation	42
S.C.	Insulin	Injection vials	Diabetes management	Subcutaneous injections	Patient compliance issues (needle phobia), frequent administration, and risk of infections	39
	Erythropoietin	Hydrogel	Anemia treatment	Thermosensitive hydrogel containing nanoparticles to avoid protein aggregation and to achieve sustained release	Frequent injection, no other patient-friendly administration routes	43
I.V.	Interferon-*α*	PEGylation solutions	Hepatitis B/C, oncology	PEGylation for extended half-life	Immune reactions, injection site pain, and protein aggregation during storage	40
Mucosal	Recombinant growth hormone (RGH)	Cell penetrating peptide (CPP) together with nanoparticle	Growth hormone deficiency	NPs improve the stability of CPP and therapeutic proteins and peptides	Poor permeation across intestinal mucosal barriers limits the oral delivery of prospective therapeutic proteins and peptides	44
Sublingual	Ovalbumin (OVA)	Mucoadhesive patch (chitosan and hyaluronic acid)	Preclinical: immunization, anti-cancer	Mucoadhesive patch allowed an extended time of contact between the protein and the mucosa	The dilution of bioactive compounds in saliva	45
Nasal	Insulin	Liposomes functionalized with cell-penetrating peptides	Anti-diabetes	CPPs are able to penetrate cell membranes, and liposomes are capable of protecting the drug from rapid elimination or degradation	Mucociliary clearance mechanism, low permeability of the nasal epithelium	46

### 2.2 Challenges in topical and transdermal delivery

**Figure 1:**
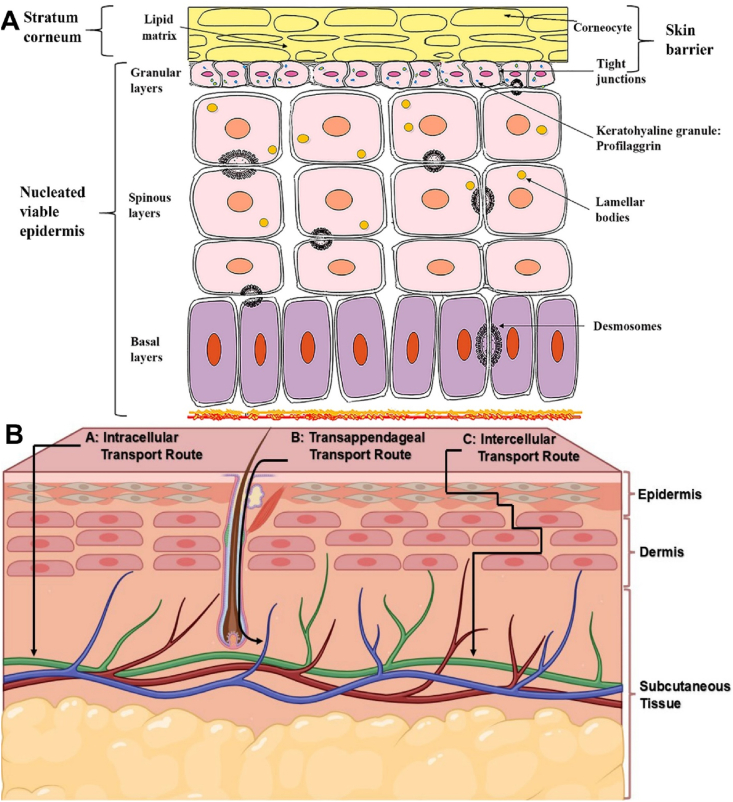
(A) Schematic representation of the skin epidermis, illustrating its distinct layers and key cellular components; (B) Overview of the three primary pathways for the delivery of active compounds through the skin: (i) intracellular (through the cells), (ii) transappendageal (*via* hair follicles and sweat glands), and (iii) intercellular (between the cells).

The skin, being the largest organ in the body, acts as the outermost protective barrier and has an average surface area of approximately 1.8 m^2^ ^48^. Structurally, the skin consists of three primary layers: the epidermis, dermis, and subcutaneous tissue, along with various appendages such as apocrine glands, sweat glands, hair follicles, and sebaceous glands^49^. The thickness of these layers varies across different anatomical sites; for example, the skin on the forehead averages 1.57 ± 0.2 mm, whereas it measures around 0.95 ± 0.11 mm on the forearm^50^. These layers, particularly the stratum corneum, play an essential part in safeguarding the body from microbial invasion and environmental insults, but also pose a significant barrier to the transdermal delivery of exogenous peptides and proteins^51^. Among these layers, the epidermis is the initial and primary obstacle that should be addressed to enable efficient drug absorption^52^. Although it comprises only about 5% of the total skin thickness, the epidermis performs vital functions such as acting as a physical barrier, producing keratin, and facilitating the renewal of corneocytes^53^. The structure of epidermis can be categorized into four parts, as shown in Fig. 1A, which are stratum corneum, granular layer, spinous layer, and basal layer. Of these four parts, the first barrier to penetration is the physical barriers imposed by the stratum corneum and tight junctions.

#### 2.2.1 Physical barriers

The stratum corneum (SC) is the skin's primary barrier, constituting the outermost, non-living layer of the epidermis, and has an approximate thickness of 15 μm^55^. It consists of around 15 layers of flattened, non-viable keratinocytes—referred to as corneocytes—which have undergone terminal differentiation, losing their nuclei and metabolic activity^55^. The SC contains roughly 5%–20% water, 40% keratinized protein, and 15% lipid content^56^. The interaction between lipids and corneocytes forms a cohesive, water-resistant barrier that limits transepidermal water loss (TEWL), maintaining skin hydration and flexibility while also protecting against the entry of pathogens and chemical substances^57^^,^^58^. This layer is highly selective and significantly impedes the penetration of hydrophilic compounds and macromolecules^59^. Only drug candidates possessing optimal physicochemical characteristics are capable of traversing this barrier to reach deeper skin layers.

Another physical barrier in dermal or transdermal delivery is tight junctions (TJs), which are specialized cell-to-cell connections found in both simple and stratified epithelial tissues, and also in endothelial layers^60^. These structures are composed of various transmembrane proteins, including members of the claudin family and TJ-associated MARVEL proteins such as occludin and junctional adhesion molecules. Additionally, intracellular scaffold proteins—commonly referred to as TJ plaque proteins—such as zonula occludens (ZO-1, ZO-2, ZO-3), MUPP-1, and cingulin, anchor these complexes to the actin cytoskeleton^61^. The principal role of TJs is to seal the paracellular space, thereby limiting the diffusion of substances between adjacent cells^62^. Beyond their barrier function, TJs also participate in cellular processes such as polarization, differentiation, proliferation, and signal transduction^63^. These junctions form a selective barrier that prevents the passage of molecules and ions of various sizes; for instance, tracer molecules with molecular weights of 557 Da, 1500 Da, 5 kDa, and 31/32 kDa, as well as ion tracers like La^3+^, are impeded by TJs in the granular layer following intradermal administration^64^. Computational models further support the role of TJs in restricting calcium ion permeability^65^. According to the pore and leak pathway model, which distinguishes between diffusion routes for small ions and larger macromolecules, epidermal TJs serve as a restrictive barrier to both classes of molecules^64^.

#### 2.2.2 Challenges in transdermal/dermal routes of transport

Permeation of compounds crossing the SC is based on a passive diffusion-driven transport. The main difficulty for the rate of diffusion is that these layers of skin have the highest resistance to penetration for most chemicals compared to other routes of administration. As for the diffusion in the layers of epidermis and dermis, the rate of penetration can be influenced by the nature of the drug (hydrophilic or lipophilic). According to the nature of the drug candidates, there are typically three pathways (Fig. 1B) in which compounds are transported into the dermis: intracellular, appendageal, and intercellular routes^54^^,^^66^. Representative examples of peptides/proteins and other compounds associated with various transdermal transport pathways are shown in Table 2^28^^,^^29^^,^^31^^,^^32^^,^^67^, ^68^, ^69^, ^70^, ^71^, ^72^, ^73^.

**Table 2:** Representative examples of peptides/proteins and other compounds associated with various transdermal transport pathways^28^^,^^29^^,^^31^^,^^32^^,^^67^, ^68^, ^69^.

Pathway	Chemical example	Type	Transport/mechanism notes	Ref.
Intracellular (transcellular *via* transcytosis)	GHRP-6 IGF-I Proapoptotic peptide KLA Platelet-derived growth factor-BB Insulin Cyclosporin A SNAC + semaglutide	Lipophilic peptides/proteins	Endocytosis-mediated lipophilic peptides/proteins	67, 68, 69
Intercellular	Acyclovir Tacrolimus Vitamin C Caffeine Curcumin analogues	Low molecular weight compounds	Diffusion is modulated by molecular weight, partition coefficient, and vehicle composition.	70, 71, 72
Appendageal (transfollicular/sweat glands)	des(1–3)IGF-I + TD-1 Nanoparticle-encapsulated peptides/proteins	Nanoparticle systems	Through follicular openings and sweat glands by particle size (<500 nm).	73

##### 2.2.2.1 Intracellular (transcellular) transport

The intracellular route, also known as the transcellular pathway, refers to the movement of solutes across a cell membrane, which includes transcellular active carrier-mediated transport, transcellular diffusion, and transcytosis^74^. Because the cell membrane is hydrophobic in nature, it may be resistant to the passive diffusion of charged or hydrophilic compounds. Active carrier-mediated transport assists certain molecules in moving across the skin barriers and against the concentration gradient by the use of energy, unlike transcellular diffusion^75^. Intracellular is the major transport pathway for macromolecules across the cell membranes^76^. These macromolecules are typically internalized into vesicles on one side of the cell, transported *via* the cytoplasm, and released on the opposite side through a process known as transcytosis^76^. Nevertheless, the majority of experimental evidence indicates that the primary route for molecular diffusion across the stratum corneum (SC) is the intercellular pathway, as outlined below.

##### 2.2.2.2 Intercellular transport

The more common transportation mechanism penetrating the SC is *via* the intercellular pathway because of the fact that cornified cells have an impermeable feature, where the external lipids of cells are important for the skin's barrier function^77^^,^^78^. These external lipids mainly consist of fatty acids, ceramides, triglycerides, and cholesterol^79^. Drugs crossing the skin *via* this route must pass these surrounding lipids, increasing their difficulty^79^. This makes the pathway of drug diffusion many times longer (450 μm) than the actual thickness of the SC (30 μm), thereby reducing the penetration rate of chemicals^80^. Studies from Matsuzaki suggested that polar compounds of molecular weight less than 500 Da have similar permeability to potassium ions, and these molecules are almost constant in permeability^54^^,^^81^. Some compounds, depending on the formulation, can cross through the SC faster than the intercellular route by using the appendageal route^82^^,^^83^.

##### 2.2.2.3 Appendageal transport

Appendageal transport primarily involves the utilization of skin appendages such as hair follicles, sweat glands, and sebaceous glands for the delivery of therapeutic agents^84^. The contribution of the appendageal route to overall transdermal drug diffusion is generally minimal, primarily due to its relatively small surface area compared to other pathways such as the intercellular route^85^^,^^86^. Moreover, the structural complexity of hair follicles presents an additional challenge, requiring a two-step process for effective delivery: initial penetration into the follicular canal, followed by *trans*-follicular transport into the viable tissue surrounding the appendages^87^. Nevertheless, research by Patzelt et al.^88^ has demonstrated that enhancing drug delivery *via* this route often necessitates modifications to the physicochemical characteristics of the therapeutic agent or the incorporation of follicular penetration enhancers^88^. This is important as nanocarrier systems have been shown to be a beneficial way to overcome some of these barriers and transport drugs *via* this route^88^.

#### 2.2.3 Biochemical barriers

##### 2.2.3.1 Antimicrobial peptides in skin

The primary chemical barrier of the skin includes antimicrobial peptides (AMPs), which are synthesized by keratinocytes and immune cells. These peptides play crucial roles in host defense, inflammation, and wound healing^89^. AMPs display broad-spectrum activity, effectively targeting bacteria, fungi, and viruses^90^. While some AMPs are constitutively expressed at low levels in healthy skin, their production can be markedly upregulated in response to infection or tissue injury^91^. Others are inducible and expressed only upon pathogenic challenge^91^. In addition to AMPs, the acidic pH of the skin surface constitutes a vital component of the chemical barrier, as it inhibits microbial proliferation^92^. Notably, AMPs may also contribute to the formation of a protein corona around nanoparticles, potentially influencing their interaction with skin tissue. Moreover, pH is a key factor influencing the function of specific drug formulations that exploit local pH variations within the skin to trigger sustained drug release^93^. It is significant to highlight that skin disorders, such as atopic dermatitis, are often associated with an elevated skin surface pH, which may compromise the effectiveness of this natural defense mechanism^94^.

##### 2.2.3.2 Enzymes in skin

Foreign substances that bypass the initial skin barrier may undergo biotransformation within the viable layers of skin^95^. This metabolic processing typically enhances the water solubility of xenobiotics, thereby facilitating their excretion and reducing systemic bioavailability^96^. Such metabolic activity may partly explain the reduced or negligible pharmacological efficacy of certain potent drugs following dermal application^97^. The primary function of cutaneous biotransformation is to detoxify and convert lipophilic molecules into more hydrophilic and less active forms for easier elimination^98^. This occurs through a two-phase enzymatic process: the functionalization phase (phase I) and the conjugation phase (phase II)^99^. During phase I, oxidative, reductive, or hydrolytic reactions introduce or expose polar functional groups on the substrate^100^. In phase II, these metabolites undergo covalent conjugation with endogenous hydrophilic molecules such as glucuronic acid, sulfate, or glycine, resulting in compounds with increased molecular weight and hydrophilicity^101^. Although this modification enhances solubility and promotes excretion, it often leads to reduced biological activity of the compound. While hepatic metabolism remains the most efficient and well-characterized system for biotransformation in the human body, the skin also expresses several enzymatic families capable of metabolizing topically applied substances^102^. However, the extent and implications of cutaneous metabolism are not yet fully understood. Table 3^97^^,^^103^, ^104^, ^105^, ^106^, ^107^, ^108^, ^109^, ^110^, ^111^ summarizes the key enzyme families identified in human skin that are involved in the degradation of transdermally delivered drugs^97^.

**Table 3:** Enzymes associated with skin and their cleavage functions^97^^,^^103^, ^104^, ^105^, ^106^, ^107^, ^108^, ^109^, ^110^, ^111^.

Name	Cleavage specificity	Example	Ref.
Alcohol dehydrogenase	Catalyzes the oxidation of aliphatic alcohols to their corresponding aldehydes	Retinol	97
Aldehyde dehydrogenase	Oxidizes these aldehydes to form carboxylic acids	Acetaldehyde	103
Carboxylesterase	Mediates the hydrolysis of ester compounds such as 4-methylumbelliferone heptanoate and acetate, yielding the fluorescent compound 4-methylumbelliferone	2-Arachidonoylgylcerol	104
Cytochrome P450	Facilitate the monooxygenation of substrates through the insertion of one oxygen atom, typically producing alcohols and water as byproducts	Testosterone	105
Flavin-dependent monooxygenase	Involved in the *N*-oxidation of secondary and tertiary amines, contributing to phase I metabolic biotransformation	Tertiary amines	106
Hydroxylases	Aromatic ring cleavage in phase I oxidation reactions of skin aging	Phenylalanine	107
5*α*-Reductase	C=C double bond cleavage in phase I reduction reactions of skin aging	Testosterone	108
Esterase	Ester bond cleavage in phase I hydrolysis reactions of skin aging	Butyrylcholine	109
Glutathione-*S*-transferases	Glutathione oxidation in phase II conjugation reactions of skin aging	Cisplatin, glutathione	110,111

##### 2.2.3.3 Immunological factors

Immunological factors involved in dermal and transdermal drug delivery encompass elements of both the cellular and humoral branches of the immune system. Key cellular components include Langerhans cells, dermal dendritic cells, mast cells, basophils, and T lymphocytes, while humoral factors primarily involve cytokines^112^. Pathogen recognition is facilitated through conserved pattern recognition receptors (PRRs), such as Toll-like receptors (TLRs) and nucleotide-binding oligomerization domain (NOD)-like receptors, which detect pathogen-associated molecular patterns (PAMPs), including lipopolysaccharides and peptidoglycans^113^. These immune components may impact transdermal drug delivery outcomes, as drug delivery systems can interact with and modulate immune cells, which in turn may affect other skin barrier mechanisms—for instance, through the release of pro-inflammatory or regulatory cytokines^97^^,^^114^.

### 2.3 Challenges in oral delivery

**Figure 2:**
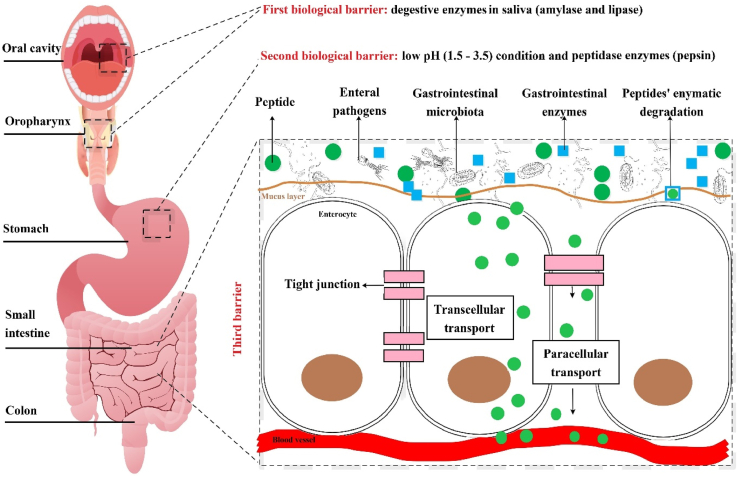
Schematic depiction of the oral administration pathway and the key barriers encountered during the delivery of therapeutic peptides and proteins.

Oral administration is widely favored owing to its ease of use and high patient adherence; however, it encounters multiple physiological and structural barriers within the gastrointestinal tract (GIT), as illustrated and summarized in Fig. 2^115^. Therapeutic peptides and proteins must cross multiple physical and biochemical barriers throughout the oral cavity, oropharynx, stomach, and intestines, before transiting through the intestinal epithelium into the bloodstream or lymphatic system^116^^,^^117^. Among these challenges for oral delivery, the most important barrier to penetration is the physical and functional barrier of the gastrointestinal mucus layer and the unstirred water layer^118^^,^^119^.

#### 2.3.1 Physical barriers

##### 2.3.1.1 Gastrointestinal mucus layer

The gastrointestinal mucus layer is a sophisticated structure that consists of cell-associated mucins and glycoproteins, positioned adjacent to absorptive enterocytes in the epithelial mucosa of the stomach and intestines. This layer functions both as a lubricant to facilitate the passage of ingested materials and as a protective barrier that captures larger molecules and pathogens^120^. Within the gastric environment, the mucus layer exhibits a pH gradient, with values dropping below two at the luminal interface and approaching neutrality at the epithelial boundary^120^. Mucin exhibits a rapid and dynamic turnover, characterized by its breakdown in the acidic and enzyme-rich gastric environment, counterbalanced by ongoing synthesis to maintain mucosal integrity. Up to 20 distinct mucin isoforms have been identified as either secreted or membrane-bound, the latter possessing short cytoplasmic domains that facilitate intracellular signaling processes^120^. Secreted mucins are cross-linked through disulfide bonds to form macromolecules that are heavily glycosylated, providing structural stability and resistance to enzymatic degradation^120^^,^^121^. The size, structure, and intermolecular interactions of mucus influence the diffusion rate of peptides, proteins, and larger molecules through this layer^120^^,^^122^. Furthermore, the mucus layer serves as a reservoir for antimicrobial peptides such as defensins and entraps microorganisms within its outer regions, thereby limiting microbial access to and interaction with the underlying enterocytes^120^^,^^123^. Certain mucins, such as MUC2, also regulate luminal bacterial colonization by modulating dendritic cell activity, which suppresses inflammatory responses and enhances tolerogenic responses within the gut mucosa^120^^,^^124^. In-depth research using both cell-based and cell-free models has elucidated key factors affecting hydrophobic drug–mucus interactions, emphasizing the importance of peptide/protein molecular weight, charge, and mucus pore size in determining mobility and penetration across experimental mucus systems^120^^,^^125^. Nevertheless, the further *ex vivo* experimental models of peptide/protein permeation through mucus accurately replicate the complexity of the *in vivo* mucosal layer remains unclear.

##### 2.3.1.2 Unstirred water layer

The unstirred water layer (UWL) in the intestine refers to a slow-moving layer composed of water, mucus, and glycocalyx, situated between the apical surface of intestinal epithelial cells and the lumen^126^^,^^127^. The UWL also exhibits a lower pH of 0.5–1.0 when compared to the equivalent region of the GI lumen^126^^,^^127^. The UWL provides a hydrophilic and viscous barrier for the intestinal absorption of all substances in addition to the aqueous-lipid interface and the lipophilic cell membrane itself^128^. The UWL over intestinal villi also decreases the potential surface area for absorption^129^. The overall charge of the UWL is negative due to the secretion of mucins^130^. This can cause repulsion towards negatively charged compounds, hence decreased absorption. The permeability of substances across the UWL is associated with influencers such as the rate of stirring, effective surface area, and thickness of the UWL^131^^,^^132^.

#### 2.3.2 Challenges in oral routes of transport across the intestinal membrane

The absorption of peptides and proteins administered orally across the intestinal membrane can occur through various pathways, including the transcellular route *via* enterocyte membranes, the paracellular route through tight junctions between epithelial cells, active carrier-mediated transport, and endocytosis^133^.

##### 2.3.2.1 Passive paracellular transport

Paracellular passive diffusion is mainly restricted by TJs. Composed of claudins and occludins, TJs are important for intercellular adhesion and maintenance of apical and basolateral polarity^134^^,^^135^. Relative to the transcellular surface area, the paracellular surface area accounts for up to 1% of the total surface area for absorption^136^. However, the paracellular pathway for transport of peptides and proteins, especially hydrophilic bioactive compounds, is important, as it can bypass the hydrophobic phospholipid bilayer. Pore size of tight junctions of rat intestinal epithelium has been reported to be between 8 and 13 Å and can allow transport of rigid spherical compounds below this limit^137^. Paracellular uptake of compounds, therefore, favors small compounds of high conformational freedom, such as peptides and proteins. Leakiness of tight junctions can be influenced by endogenous and exogenous factors such as permeation enhancers^138^.

##### 2.3.2.2 Passive transcellular transport

The transcellular passive diffusion route across the cell membrane is dependent on the permeability of both the apical and basolateral membranes as well as the physical properties of peptides and proteins^139^^,^^140^. Transcellular diffusion favors more hydrophobic compounds, as the lipid bilayer of cell membranes is hydrophobic^1^. Small peptides are generally hydrophilic, so uptake of peptides *via* the transcellular route is proposed to occur through water channels formed in the cell membrane, allowing passage of solutes with minimum interaction with the phospholipid bilayer^141^^,^^142^. In addition to the concentration gradient and membrane permeability, blood flow, GI transit time, and surface area are other physiological factors that can also affect the rate of uptake for the transcellular passive diffusion route^141^^,^^143^.

##### 2.3.2.3 Carrier-mediated transport and endocytosis

Carrier-mediated transport is the main route of uptake for small di-/tri/tetra/pentapeptides such as GSH and thymopentin (TP5) through the mammalian intestine^144^. Utilizing a pre-established proton gradient and membrane potential set up by the Na^+^/H^+^ exchanger, peptides are able to be absorbed into cells along with protons using peptide carriers such as the peptide transporter located on apical membranes^145^^,^^146^. However, due to the lack of specific transporter carriers on the basolateral side and the abundance of cytosolic peptidases in intestinal epithelium cells, 90% of absorbed peptides are broken down into amino acids before leaving the basolateral membrane^147^.

Endocytosis is an active transport mechanism for the absorption of material into cells^148^. Endocytosis is involved in the formation of enclosed vesicles, entrapping extracellular material with extracellular fluid, and can be either receptor-mediated or non-receptor-mediated^149^. Substances taken up by endocytosis can optionally interact with the endosome membrane. This allows a lot of flexibility with the compounds it can uptake^150^. Pinocytosis is a form of non-receptor-mediated endocytosis, which can indiscriminately incorporate extracellular fluids and solutes, making it a viable strategy for the absorption of small hydrophilic peptides^149^^,^^150^. Phagocytosis is another form of endocytosis, which may incorporate extracellular solid debris, as well as microorganisms^151^. Finally, receptor-mediated endocytosis is another pathway for absorption of peptides and larger proteins, but is only initiated if membrane-bound surface proteins are triggered^152^. Common membrane receptors that can trigger endocytosis are caveolin and clathrin^153^. Specific moieties that trigger these receptors can be conjugated to peptides and proteins to trigger receptor-mediated endocytosis, such as the cell-penetrating peptide polyarginine chain^154^.

#### 2.3.3 Biochemical barriers

##### 2.3.3.1 Peptide-degrading enzymes in the oral route of administration

Upon ingestion, peptide formulations are first exposed to digestive enzymes such as amylase and lipase in the saliva. Once they enter the stomach, they encounter a highly acidic environment and enzymes like pepsin and cathepsin, which are efficient in breaking down peptides. In the small intestine, additional proteolytic enzymes, including those secreted by the pancreas and brush border membrane peptidases expressed by enterocytes, continue the digestion process. These enzymes include trypsin, chymotrypsin, carboxypeptidase, and various dipeptidases and aminopeptidases^155^. Models commonly used to study drug transport across epithelial cells may not fully replicate the complex enzymatic conditions, including regional pH variations within the gut lumen, potentially leading to an overestimation of peptide bioavailability^120^. Table 4 shows a summary of the activity of endo and exopeptidases associated with the cleavage of peptides *via* the oral route of administration^152^^,^^156^, ^157^, ^158^, ^159^, ^160^, ^161^, ^162^, ^163^, ^164^, ^165^, ^166^, ^167^.

**Table 4:** Associated enzymes in the oral route of administration and their cleavage functions^152^^,^^156^, ^157^, ^158^, ^159^, ^160^, ^161^, ^162^, ^163^, ^164^, ^165^, ^166^, ^167^.

Name	Cleavage specificity	Example	Ref.
Pepsin	Low specificity and preference for before non-terminal Leu, Phe, Trp, or Tyr, except adjacent to pro	Casein, collagen	156, 157,158
Trypsin	After non-terminal Arg or Lys except adjacent to pro	Bovine serum albumin, insulin	157,159
Elastase	Carboxylesterase catalyzes the ester hydrolysis of 4-methylumbelliferone heptanoate and acetate, converting them into 4-methylumbelliferone	Elastin peptides	152,157
Endopeptidase	Low specificity before non-terminal amino acids	Enkephalins	160
Dipeptidyl peptidase I	Low specificity and preference for after the second amino acid from the N-terminus, except for N-terminal Arg or Lys	Proinsulin	161
Dipeptidyl peptidase IV	Low specificity and preference for after the second amino acid from the N-terminus if Ala or pro	Glucagon-like peptide-1 (GLP-1)	161
*N*-Acyl-amino acid releasing enzyme	High specificity and cleaves N-terminal *N*-acetyl amino acids	*N*-Acetylaspartate	162
Pyroglutamate aminopeptidase	High specificity and cleaves N-terminal cyclic lactam amino acids	Pyrrolidone carboxylate peptides	167
Methionine aminopeptidase	High specificity and cleaves N-terminal Met	Adenylate cyclase	157,163
Aminopeptidase (A, B, W, and II)	High specificity and cleaves N-terminal amino acids	Leukotrienes	157,164
Aminopeptidase (N and P)	Low specificity and cleaves N-terminal amino acids	Neuropeptide Y	157,164
Cytosol alanyl aminopeptidase	Low specificity and preference for N-terminal Ala	Ala-derivatives of peptides	157,164
Tripeptide aminopeptidase	After the third amino acid from the N-terminus	Glutathione, bradykinin	157,164
Angiotensin-converting enzyme	After the second amino acid from the C-terminus	Angiotensin I	165
Carboxypeptidase (A, B, M, and P)	High specificity and cleaves C-terminal amino acids	Insulin	166

##### 2.3.3.2 Sulfur barrier

The sulfur barrier in oral drug delivery refers to the influence of sulfur-containing biological components on drug absorption, metabolism, and bioavailability within the gastrointestinal (GI) tract^168^. This barrier is primarily attributed to sulfur-rich macromolecules such as mucin glycoproteins, which form a protective mucus layer that hinders drug diffusion, and enzymatic systems including sulfotransferases (SULTs) and glutathione (GSH)-mediated metabolism, which contribute to drug biotransformation and clearance^169^. Sulfation, a key phase II metabolic pathway, often leads to rapid drug inactivation and elimination, particularly for hydrophilic and peptide-based drugs, thereby reducing their systemic availability^170^. Furthermore, interactions with sulfur-containing amino acids such as cysteine and methionine can impact drug stability and absorption^171^. Strategies to overcome the sulfur barrier include prodrug design, which enhances drug stability and transport before metabolic activation, nanocarrier systems that shield drugs from premature metabolism, and enzyme inhibitors that modulate sulfur-mediated metabolism. Understanding and addressing the sulfur barrier is crucial for optimizing the oral bioavailability of peptide drugs, biologics, and small-molecule therapeutics, particularly those prone to extensive sulfation or glutathione conjugation.

##### 2.3.3.3 Inter-individual variability

A critical factor in formulating orally delivered peptide and protein therapeutics lies in the substantial variability in gastrointestinal tract physiology among individuals. This includes differences in mucus and enzyme production, as well as variations in gastric emptying and gut motility^120^^,^^172^. The rate of gastrointestinal transit is a key determinant of epithelial exposure to ingested peptides, and it can be influenced by factors such as meal composition, meal size, meal timing, and age. Variations in gut motility and absorption rates are particularly important when developing therapeutics like insulin for diabetes. Individuals with mild dysglycaemia or diabetes often exhibit dysregulated gut motility, and impaired gastric emptying is commonly observed in individuals with type 1 diabetes, which can further complicate glycaemic control^120^^,^^173^. Additionally, differences in the luminal epithelial environment, such as the expression levels of digestive enzymes, peptidases, and transporters, can influence the timing and extent of transmucosal drug absorption^120^^,^^174^.

#### 2.3.4 Challenges in oral delivery across the blood‒brain barrier

##### 2.3.4.1 Blood‒brain barrier

The blood‒brain barrier (BBB) poses both physical and enzymatic barriers that restrict the passage of various compounds, including numerous peptides and proteins^175^. The BBB is formed by brain capillary endothelial cells, pericytes, astrocytes, and neuronal cells, and performs several vital functions. These involve preserving neural connectivity, maintaining ion homeostasis, preventing the entry of neurotoxic molecules, and selectively transporting essential nutrients into the brain^175^^,^^176^. A key feature of the BBB is the asymmetrical distribution of membrane-bound transport systems across the apical and basolateral surfaces of the endothelial cells. Notably, P-glycoprotein efflux pumps located on the apical surfaces play a crucial role in protecting the brain from potentially harmful or unwanted compounds^176^^,^^177^. The endothelial cells of brain capillaries are tightly joined by continuous TJs, which, together with adherens junctions on the apical side, form a robust physical barrier that restricts paracellular transport into the brain^177^^,^^178^. These tight junctions are associated with exceptionally high transendothelial electrical resistance, rendering the BBB highly resistant to passive diffusion^179^. As a result, approximately 100% of macromolecular drugs and 98% of small lipophilic drug candidates (500 to 1000 Da) are unable to cross the BBB, with the exception of small lipophilic molecules (<500 Da) such as certain nutrients^180^^,^^181^. Furthermore, an additional metabolic barrier exists within the BBB due to the presence of various proteolytic enzymes, both intracellularly and extracellularly^179^^,^^180^. Collectively, these characteristics make the BBB a significant challenge for drug delivery. As a result, researchers are increasingly exploring the use of ligands to target specific transporters or carrier systems, aiming to improve the uptake of therapeutic compounds penetrating the BBB, as summarized in Table 5^180^^,^^182^, ^183^, ^184^, ^185^, ^186^, ^187^.

**Table 5:** Brief list of peptide and protein delivery penetrating the BBB^180^^,^^182^, ^183^, ^184^, ^185^, ^186^, ^187^.

Peptide/protein	Strategies	Route of administration	Therapeutic effect	Ref.
Leucine-enkephalin	*N*-Palmitoyl *N*-monomethyl, *N*-dimethyl, *N*-trimethyl-6-*O*-glycolchitosan ligand	Oral	Antinociception	182
Neurotensin	Angiopep-2 ligand	IV	Allodynia	183
*O-*palmitoyl tyrosinate ester-dalarg	Nanofibers	IV	Antinociception	180
Dalargin	Polymeric nanoparticles	IV	Antinociception	184
Opioid peptide	VH0445 peptide conjugates	IV	Antinociception	185
4-Demethyl penclomedine	Derivative of 4-demethyl penclomedine conjugation	IV	Brain neoplasms	186
Aurimmune (TNF-*α* conjugate)	Cytokine protein chemical conjugation	IV	Solid tumor	187

**Figure 3:**
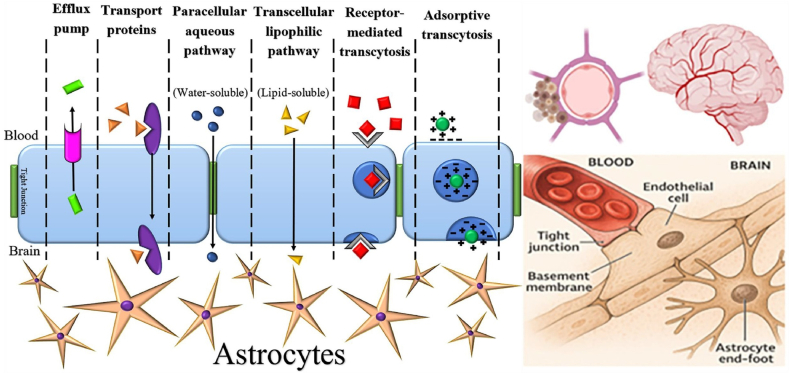
Schematic representation of the various drug transport routes across the blood–brain barrier.

The main mechanisms that enable drug transport penetrating the BBB include the receptor-mediated transcytosis, transcellular pathway, adsorptive transcytosis, and transporter protein-mediated transport, as depicted in Fig. 3^180^. Any ligand or chemical modification aimed at improving drug delivery across the BBB is integrated into the transport mechanisms presented in Fig. 3. The efficiency of the paracellular and transcellular lipophilic pathways, as well as adsorptive transcytosis, is affected by the physical and chemical properties of the drug or ligand, particularly its lipophilicity and ionization state, which determine its interaction with the BBB. In contrast, transport *via* receptor-mediated transcytosis, transporter proteins, and efflux pumps is primarily governed by the affinity between the receptor and the drug or ligand, facilitating its uptake across the barrier, as depicted in Fig. 3. The enzymatic barrier further complicates drug delivery to the brain due to the presence of numerous proteolytic enzymes on the endothelial surface, which can degrade therapeutic molecules before they reach their target site. However, researchers have achieved some success in BBB penetration by exploiting specific receptors that are overexpressed on the endothelial cells of the BBB. Notably, various receptors, including those for transferrin, insulin, low-density lipoprotein-related proteins, diphtheria toxin, heparin-binding EGF-like growth factors, and leptin, have been investigated as potential targets for facilitating drug delivery across the blood–brain barrier^175^^,^^176^. Additionally, transport proteins for glutathione and choline, which are highly expressed at the BBB, have also been investigated as potential transport mechanisms^176^^,^^177^. To improve BBB penetration, two primary strategies have been developed: (1) the use of active targeting ligands and (2) the application of cell-penetrating peptides (CPPs)^188^^,^^189^. To further improve brain uptake and specificity, scientists have increasingly designed multifunctional delivery systems that incorporate multiple ligands within a single formulation, allowing for enhanced targeting and transport efficiency.

##### 2.3.4.2 Brain targeting ligands

A promising non-invasive approach for improving oral drug delivery to the brain is the use of active targeting ligands that specifically interact with the BBB, as outlined in Table 6^180^^,^^190^, ^191^, ^192^, ^193^, ^194^, ^195^, ^196^, ^197^, ^198^, ^199^. These ligands are equipped with functional groups that selectively bind to receptors on brain endothelial cells, thereby promoting transport across the BBB. This transport occurs through mechanisms including receptor-mediated transcytosis, carrier-mediated transport, or adsorptive-mediated endocytosis. Despite their potential, several challenges hinder the effective use of brain-targeting ligands. Studies have shown that dual-ligand functionalization of drug delivery systems significantly improves BBB penetration compared to single-ligand approaches. However, optimizing ligand selection remains critical, as the protective role of drug carriers reduces the necessity for enzyme inhibitors to prevent peptide degradation. A major limitation is the distinct physiological differences between the GIT and the BBB. A single ligand or transport mechanism is typically insufficient for facilitating absorption across both barriers due to their structural and functional disparities. Even when a receptor is expressed on both the GIT and BBB, its utilization for both pathways *via* a single ligand is often suboptimal due to variations in receptor density, localization, and function. Furthermore, even with direct intravenous administration, brain uptake remains minimal, underscoring the difficulty of bypassing the BBB. Future oral drug delivery strategies must therefore incorporate tailored approaches that address the challenges of both the GIT and the BBB independently to achieve efficient brain-targeted therapy.

**Table 6:** Examples of different types of ligands for receptor-mediated transport in the BBB^180^^,^^190^, ^191^, ^192^, ^193^, ^194^, ^195^, ^196^, ^197^, ^198^, ^199^.

Ligand	Target receptor	Transport mechanism	Advantages	Challenges	Key findings	Ref.
Transferrin	Transferrin receptor (TfR1, TfR2)	Receptor-mediated transcytosis	Well-studied; involved in iron homeostasis	High endogenous transferrin competes for receptor sites	Transferrin-PEGylated nanoparticles delivered ∼20% dose to the brain	190
Monoclonal antibodies (OX26, 8D3, RI7)	Transferrin receptor (TfR)	Receptor-mediated transcytosis	High affinity to receptor; effective brain uptake	Species-specificity (OX26 for rats, RI7 for humans)	RI7 ligand showed the highest brain selectivity (∼3.1% uptake)	191
Lactoferrin	Lactoferrin receptor	Receptor-mediated and adsorptive transcytosis	Lower plasma competition; unidirectional transport	Receptor variability across species and tissues	Lactoferrin-modified nanoparticles showed superior BBB penetration	192
Insulin	Insulin receptor (IR)	Receptor-mediated transcytosis	High brain uptake potential	Short half-life; risk of hypoglycemia	MAb 83-14 delivered ∼4% dose to brain in primates; humanized MAbs reduce immunogenicity	193
Low-density lipoprotein (LDL) & angiopep-2	LDL receptor (LDL-R, LRP-1)	Endocytosis	High transcytosis and brain accumulation	Cargo-dependent efficacy; variable receptor expression	Angiopep-2 demonstrated superior transport *vs*. transferrin and lactoferrin	194
Glucose	GLUT1 (glucose transporter 1)	Carrier-mediated transport (CMT)	Essential nutrient transport system	High endogenous glucose competes for transport	GLUT1-conjugated nanoparticles improved brain uptake	195
Leptin	Leptin receptor (OB-R)	Receptor-mediated transcytosis	Naturally crosses the BBB; hormone-mediated pathway	Receptor saturation limits dose delivery	Leptin-functionalized nanocarriers showed increased brain permeability	196
Apolipoprotein E (ApoE)	LDL receptor (LDL–R, LRP–1)	Receptor-mediated transcytosis	Endogenous brain transport system; high targeting efficiency	Competitive inhibition with natural ApoE	ApoE-coated nanoparticles significantly enhanced BBB penetration	197
Tetanus toxin C fragment (TTCF)	Gangliosides (GT1b, GD1b)	Adsorptive-mediated transcytosis (AMT)	High neuronal affinity; retrograde transport	Potential immunogenicity	TTCF-modified nanocarriers improved neuron-specific delivery	198
Rabies virus glycoprotein (RVG29)	Nicotinic acetylcholine receptor (nAChR)	Receptor-mediated transcytosis	High specificity for neurons	Limited receptor expression in non-neuronal cells	RVG29-decorated nanoparticles showed enhanced brain targeting	199

### 2.4 Issues in parenteral delivery

**Figure 4:**
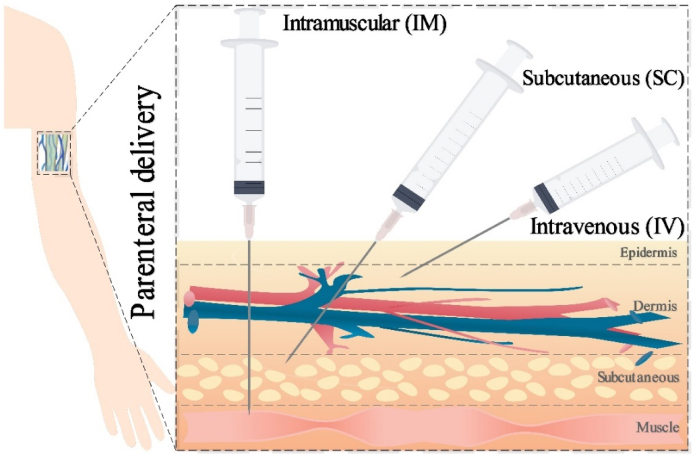
Illustration of the parenteral delivery including intramuscular (IM), subcutaneous, and intravenous (IV) injections.

Traditionally, the parental route has been the most practical and efficient way of delivering peptide therapeutics. However, parental drug delivery is painful and often requires repeated injections because of the short plasma half-life of peptides, enzymatic degradation in the blood or tissue fluid, and immunogenicity, which results in poor patient compliance. Parenteral administration of peptides is usually achieved *via* intravenous (IV), subcutaneous (SC), or intramuscular (IM) injections, as shown in Fig. 4^200^.

#### 2.4.1 IV injection

The main challenges of IV injection can be displayed in terms of physical barriers, sterilization requirements, frequent applications, and low patient compliance^201^. Therapeutic peptides administered intravenously have to cross capillary endothelial barriers to reach various organs for local or systemic applications if the target of the drug is not in the blood^202^. The structural properties and permeability of the capillary endothelium vary in different organs and are classified as continuous, fenestrated, and sinusoidal^203^, ^204^, ^205^. Continuous endothelium is present in muscles, lungs, heart, the central nervous system, and connective tissues^203^, ^204^, ^205^. The capillary endothelium in the brain (BBB) is characterized by tight junctions between cells and the absence of any gaps or fenestrations^206^. This makes brain capillaries impermeable to polar molecules. However, there are abundant mitochondria and enzymes that facilitate active transport of molecules in the brain^206^. Most of the continuous capillaries in the peripheral tissues (other than the brain) are permeable to peptides and proteins up to 70-kDa molecular weight^207^. Fenestrated endothelium is present in intestinal mucosa, renal glomeruli, and endocrine and exocrine glands^203^, ^204^, ^205^. Fenestrations are openings of 60 to 80 nm with or without diaphragm membranes and allow rapid movement of fluid and low molecular weight solutes in the respective organs^208^. Fenestrae with diaphragm membranes are impermeable to macromolecules, whereas those without diaphragms allow permeation of large macromolecules, peptides^209^. Sinusoidal capillary endothelium is found in the liver, spleen, and bone marrow^210^, ^211^, ^212^. These capillaries have large pores (size >100 nm) and do not offer a barrier to the passage of macromolecules, peptides, and proteins^213^.

Another challenge for IV injections or even all parenteral formulations is the requirement of a higher standard of sterilization^214^. However, due to the sensitive and fragile properties of peptides, the process of sterilization is special and needs extra attention. Sterile filtration is used to ensure sterility rather than a mediated approach, which directly increases the economic burden for health researchers and patients^215^. Frequent injections derived from the short half-life property of therapeutic peptides are another issue that impede the development of IV injection^216^. Meanwhile, frequent injections not only cause pain to patients but also raise the issue of inconvenience and higher cost of customer service^216^. SC and IM injections are introduced to reduce the frequency of IV injections and favor a controlled release profile to achieve a sustained action.

#### 2.4.2 SC and IM injections

Similarly, the physical barrier associated with SC and IM injections is the capillary endothelial membrane for the absorption of peptides and proteins. Small molecular weight peptides are mainly absorbed through the blood capillaries, whereas large peptides (>16 kDa) are taken up by the lymphatic system^217^. However, because of the slow rate of absorption in subcutaneous and IM injections, the difference between subcutaneous and IM with IV injections is that the injected volume at subcutaneous and IM sites is up to 2 and 5 mL, respectively^218^. This small capacity, therefore, restricts the application of high doses of therapeutic peptides. It can also result in local tissue inflammation and skin irritation and can increase the risk of reactogenicity^219^^,^^220^. For example, blood clotting factor VIII has extremely low bioavailability *via* subcutaneous because of proteolytic degradation in lymphatic fluids and phagocytosis in lymph nodes^221^.

### 2.5 Challenges in other routes of administration

Various alternative routes, including pulmonary, nasal, and buccal administration, are being explored for peptide and protein therapeutics. While these methods aim to enhance bioavailability and patient compliance, each presents significant challenges, including enzymatic degradation, limited permeability across biological membranes, and variability in absorption. Overcoming these barriers remains a critical focus in peptide drug delivery research.

The pulmonary route offers several advantages for peptide delivery, including a large surface area (∼70–100 m^2^) and a dense network of capillaries surrounding the alveoli, which facilitates rapid systemic absorption while bypassing hepatic first-pass metabolism^222^. The thin alveolar epithelium (0.1–0.2 μm) enables efficient diffusion of therapeutics into the bloodstream^223^. However, peptides and proteins delivered *via* this route face several challenges. Enzymatic degradation by pulmonary proteases, macrophage uptake, mucociliary clearance in the upper airway, and surfactant-mediated denaturation can significantly reduce bioavailability^222^. Furthermore, successful alveolar deposition is highly dependent on particle characteristics, especially aerodynamic diameter. Ideal deposition occurs with particles sized between 0.5 and 3 μm, while larger particles are likely to be deposited in the oropharyngeal region, and smaller particles may be exhaled^224^. Despite these favorable anatomical and physiological features, the systemic bioavailability of peptides delivered *via* this route remains below 50%^222^.

The nasal route presents a promising non-invasive alternative for peptide and protein delivery due to its high vascularization and relatively large absorptive surface area (∼150 cm^2^), which facilitates rapid absorption and potential brain targeting *via* olfactory and trigeminal pathways^225^. However, several physiological and biochemical barriers restrict its effectiveness. First, the nasal cavity's tight epithelial junctions limit the paracellular transport of hydrophilic and high-molecular-weight peptides, which must instead rely on less efficient transcytosis or endocytosis mechanisms^226^. Second, the nasal environment presents a hostile milieu: enzymatic degradation by nasal peptidases, acidic pH, and rapid mucociliary clearance (with turnover times of 15–20 min) can lead to premature drug elimination^227^^,^^228^. Additionally, the volume that can be administered intranasally is limited (∼150–200 μL per nostril), restricting the achievable therapeutic dose. These anatomical constraints, coupled with physicochemical challenges such as peptide hydrophilicity, instability, and limited permeability, significantly impact absorption efficiency^226^.

Buccal delivery is another alternative route under investigation for peptide administration. The buccal mucosa is relatively permeable and has lower enzymatic activity compared to the gastrointestinal tract, reducing peptide degradation^229^. Its rich blood supply enables direct systemic absorption while avoiding first-pass hepatic metabolism. However, the main limitation lies in the stratified squamous epithelial barrier, which restricts passive diffusion primarily to small, lipophilic molecules^230^. Additionally, the continuous production of saliva (∼1–1.5 L/day) can dilute the peptide concentration, while involuntary swallowing may reduce drug residence time at the site of absorption. These challenges, combined with the low intrinsic permeability of large hydrophilic peptides and proteins, have limited the translation of buccal peptide formulations beyond preclinical development^230^.

## 3 Strategies to overcome the challenges of peptide/protein delivery

To enhance the transdermal, topical, and oral delivery of peptides, overcoming physiological barriers to transport across the skin and gastrointestinal tract is crucial. Four primary strategies have been proposed to address these challenges: (1) the utilization of physicochemical penetration enhancers to increase permeability; (2) chemical modification of peptide structures to enhance lipophilicity and improve membrane interactions; (3) the incorporation of enzymatic inhibitors to improve the stability of protein and peptide drugs, particularly for oral administration; and (4) advanced formulation approaches, including nanocarrier systems, to facilitate transport and provide enzymatic protection.

### 3.1 Chemical penetration enhancers

#### 3.1.1 Chemical penetration enhancers for transdermal delivery

Chemical penetration enhancers enhance the skin permeability of molecules by interacting with the lipidic complex in the skin barriers. They achieve this by fluidizing the lipid structure and disrupting the organized lipid domains, thereby facilitating the passage of molecules. Additionally, these enhancers can influence intercellular factors, metabolic processes, and thermodynamic properties, further promoting the transdermal delivery of active agents^231^. Chemicals, including dimethylsulphoxide^232^, azone^233^, pyrrolidones^234^, fatty acids^235^, fatty alcohols^236^, and surfactants^237^ are some examples of chemical enhancers currently used in the pharmaceutical field. These are used mainly to improve the penetration of the epidermis. For example, Magnusson et al.^238^ reported a significant enhancement in the transdermal delivery of thyrotropin-releasing hormone *in vitro*, with skin penetration increasing from 0.92 ± 0.03 to 1.6 ± 0.02 μg/cm^2^·h when penetration enhancers, such as ethanol and cineole, were used. However, the effectiveness of chemical penetration enhancers in oral drug delivery is often limited by their large molecular size, which restricts their ability to diffuse across biological membranes efficiently. Additionally, the hydrophilic nature of most peptides and proteins presents a significant challenge, as their poor lipid solubility hinders passive permeability through the intestinal epithelium. These factors collectively diminish the efficacy of penetration enhancers in enhancing the bioavailability of peptide- and protein-based drugs^238^. Additionally, skin irritation is a potential concern associated with chemical enhancers, which may signal skin toxicity due to their physicochemical properties^239^.

#### 3.1.2 Penetration enhancers for oral delivery

Chitosan is a biodegradable, non-toxic polymer derived from the deacetylation of *N*-acetylglucosamine units in chitin, typically through hydrolysis under alkaline conditions at elevated temperatures^240^. Penetration enhancers represent a diverse group of chemical agents that facilitate the permeation or transport of molecules across biological membrane barriers ^241^. These enhancers operate through various mechanisms, including altering membrane fluidity, reducing mucus viscosity, promoting protein leakage across membranes, and inducing the opening of tight junctions^242^. Chitosan is a semi-natural bioadhesive polymer. The phenomenon of bioadhesion enables a higher concentration of drug to remain at the target site, thereby enhancing the therapeutic effect^243^. Mucoadhesive polymers, which adhere to the mucin layer of the mucosal epithelium, have the potential to improve the oral bioavailability of protein and peptide-based therapeutics^244^. Therefore, chitosan, owing to its penetration enhancement, has generated significant interest. For example, the chitosan-ethylenediaminetetraacetic acid (EDTA) conjugate has been shown to protect peptide and protein drugs from enzymatic degradation in the gastrointestinal tract^245^. Additionally, novel chitosan derivatives are being explored to enhance permeation, including chitosan-thiobutylamidine^246^, *N*-trimethyl chitosan^247^, and methyl-pyrrolidinone chitosan^248^. Chitosan and its derivatives are generally considered non-toxic^249^ However, several studies have highlighted potential risks associated with penetration enhancers, including the possibility of tissue damage or systemic absorption due to their low molecular weight, which could result in unintended systemic effects^250^, ^251^, ^252^.

Another prominent chemical enhancer of the successful clinical translation is the use of salcaprozate sodium (SNAC), a transient permeability enhancer that facilitates the absorption of peptide drugs across the gastrointestinal epithelium^253^. SNAC acts by temporarily modulating the local environment within the stomach or intestine, increasing membrane fluidity and enabling transcellular transport without permanently disrupting epithelial integrity^254^. Importantly, SNAC also protects peptides from proteolytic degradation by modifying pH and enzyme accessibility^255^. This approach has culminated in the approval of oral semaglutide (Rybelsus®), the first oral glucagon-like peptide-1 receptor agonist (GLP-1RA) for type 2 diabetes, by the FDA and EMA^256^. Oral semaglutide combines SNAC with a high-dose peptide formulation to achieve therapeutic plasma concentrations. Clinical trials, such as PIONEER 1–12, have demonstrated that oral semaglutide is not only efficacious in glycaemic control but also shows cardiovascular and weight-reducing benefits comparable to its injectable counterpart^257^. These advances reflect a paradigm shift in peptide drug development, demonstrating that oral delivery is becoming an increasingly viable and promising approach.

### 3.2 Physical penetration enhancers

#### 3.2.1 Electromagnetic and electrical-based methods

Iontophoresis involves the application of a mild electric current to drive charged peptides and proteins across epithelial barriers. In transdermal delivery, it enhances penetration through the stratum corneum by electrorepulsion and electroosmosis^258^. Raiman et al.^69^ demonstrated effective delivery of luteinizing hormone-releasing hormone (LHRH) and Nafarelin through human skin, reporting that pulsed direct current (DC) led to 9.87 ± 4.91 μg/cm^2^·h of LHRH delivery, while alternating current (AC) was ineffective^259^. Electroporation, which uses short high-voltage pulses, creates transient aqueous pores in lipid bilayers, facilitating the transport of macromolecules such as peptides through both skin and intestinal epithelium^260^. Its transient and localized nature makes it applicable to multiple routes, although skin remains the primary target in clinical development.

#### 3.2.2 Mechanical methods: microneedles and device-enabled capsules

Microneedles (MNs) are one of the most promising physical strategies for bypassing epithelial barriers. In transdermal delivery, solid, coated, or dissolving MNs breach the stratum corneum and deliver biomolecules into the viable epidermis or dermis^261^. For oral delivery, MN capsules function analogously, penetrating the intestinal mucosa and releasing drugs directly into underlying tissues^262^. Delivery of adalimumab using such oral MN capsules demonstrated ∼50% bioavailability in swine, indicating successful bypass of enzymatic and transport barriers^263^^,^^264^. Both approaches depend on microneedle design (*e.g.*, material, length, dissolution profile) and are influenced by local anatomical factors such as vascular density, mucus thickness, and mechanical peristalsis (in the GI tract) or skin elasticity (transdermal)^265^.

#### 3.2.3 Ultrasound (sonophoresis and sono-permeabilization)

Low-frequency ultrasound (20–100 kHz) enhances drug delivery by inducing cavitation and acoustic streaming, which disrupts tight junctions and increases tissue permeability. In transdermal delivery, ultrasound (sonophoresis) can fluidize stratum corneum lipids, while in oral delivery, it promotes mucosal uptake by increasing paracellular transport^266^. Schoellhammer et al.^265^ applied ultrasound to porcine colonic epithelium, demonstrating a 2- to 10-fold increase in the uptake of peptides and 3 to 70 kDa dextran molecules, which was not achievable without sonication^267^, ^268^, ^269^. Importantly, this enhancement was reversible and localized, indicating clinical applicability for site-specific oral delivery devices^267^^,^^268^^,^^270^.

### 3.3 Enzymatic inhibitors

Enzymatic inhibitors, as their name implies, function by inactivating specific enzymes. The co-administration of enzyme inhibitors targeting GI peptidases, enzymes responsible for metabolizing peptide drugs, can help suppress the degradation of peptides in the GI tract, thereby enhancing the oral bioavailability of peptide therapeutics^271^. Enzyme inhibitors used in conjunction with peptide drugs can be categorized into four main types: polypeptide protease inhibitors, peptide-based inhibitors, amino acid inhibitors, and non-amino acid-based inhibitors^272^.

Maianti et al.^139^ were able to observe a decrease in insulin degradation when a protease inhibitor was co-administered with insulin, resulting in an improved oral bioavailability^273^. This study demonstrated an improved reduction in plasma glucose levels and a marked increase in blood insulin concentrations at 20- and 135-min after oral insulin administration, when co-administered with a peptidase or proteinase inhibitor, in both lean and obesity-induced diabetes rat models. Bacitracin, a known enzyme inhibitor, has been utilized to inhibit the breakdown of several therapeutic peptides, such as insulin and buserelin^274^^,^^275^. Aminoboronic acid derivatives, which are amino acid-based enzyme inhibitors, have been utilized in the past to improve the delivery of peptide and protein drugs, though their use has declined in favor of more effective alternatives^276^. In contrast, polypeptide protease inhibitors have gained significant attention as adjuncts for overcoming the enzymatic barrier in the oral administration of therapeutic proteins, owing to their low toxicity and potent inhibitory activity^277^. Aprotinin, a small protein extracted from bovine pancreatic trypsin, is commonly used as a peptidic enzyme inhibitor to enhance the bioavailability of insulin^278^. For instance, Kraeling et al.^153^ achieved a 6.2% oral bioavailability of insulin using aprotinin, up from 5.0% with the same formulation without aprotinin^279^. However, a limitation of this approach is that enzyme inhibition may lead to undesirable side effects, including impaired digestion, systemic toxicity, and pancreatic hyperplasia^279^. In addition, several oral enzymatic inhibitors are currently in late-stage clinical development for insulin delivery. Notably, Oramed Pharmaceuticals has advanced oral insulin formulations incorporating protease inhibitors and absorption enhancers into phase II/III clinical trials^280^.

### 3.4 Chemical modification

Chemical modification involves the conjugation or integration of chemical moieties with peptide drug candidates to enhance their pharmacokinetic properties. The modifications can result in a new compound that mimics the original in the form of analogues. For peptide-based therapeutics, the incorporation of lipophilic moieties, such as myristic acid^281^ mystic acid^282^, lauric acid^282^, palmitic acid^282^, stearic acid^283^, and oleic acid^283^, has been widely employed to improve permeability across the skin and GI epithelial barriers. These modifications facilitate enhanced transmembrane transport and absorption, addressing one of the primary challenges associated with peptide drug delivery^284^. Extensive research has demonstrated that chemically modified peptides exhibit improved absorption and bioavailability. For instance, the bioavailability of interferon-alpha (IFN-*α*) increased from 0.4 ± 0.2 to 2.1 ± 1.1 μg/cm^2^ following fatty acylation, representing a fivefold enhancement^285^. Another notable example is Matrixyl® 3000^286^, a chemically engineered compound comprising palmitoyl oligopeptide (Pal–GHK) and palmitoyl tetrapeptide-7. Pal–GHK, synthesized through the conjugation of palmitic acid with an oligopeptide sequence, enhances lipophilicity and facilitates improved dermal absorption. Palmitoyl tetrapeptide-7, a peptidic conjugate consisting of palmitic acid and a tetrapeptide sequence (glycine–glutamine–proline–arginine), has shown notable efficacy in both cosmetic and therapeutic applications^287^.

Strategic chemical modifications introduced at enzymatic cleavage sites significantly enhance the *in vivo* stability of peptide therapeutics by preventing rapid degradation. Thus, the identification of enzymatically labile sites within the peptide sequence is critical for rational chemical modification strategies. These chemically modified peptides incorporate diverse functional groups, as detailed in the following section and summarized in Table 7 288, 289, 290, 291, 292, 293, 294.

**Table 7:** Summary of chemical modifications of peptides^288^, ^289^, ^290^, ^291^, ^292^, ^293^, ^294^.

Modification type	Description	Impact/benefits	Examples/applications	Ref.
N*-* and C*-*terminal modifications	Modification of N- and C*-*termini to protect from exopeptidase degradation (*e*.*g*., acetylation, amidation)	Improves enzymatic stability and half-life. Some modifications increase lipophilicity and membrane permeability	N-terminal acetylation of somatostatin, melanocyte-releasing hormone (*α*-MSH), TRH. *N*-Linked lipidation	288, 289, 290
Cyclization	Formation of cyclic peptides *via* peptide bonds or other bridges (*e*.*g*., disulfide, ester, ether)	Reduces conformational flexibility, increases stability, potency, selectivity, and bioavailability	Cyclic analogues of vasopressin, opioid peptides; disulfide bridge cyclization	291
Methylation of amide nitrogen	Methylation of amide nitrogen to prevent enzymatic degradation	Increases stability and resistance to proteolytic enzymes while retaining biological activity	*N*-Methylated analogues of enkephalin, substance P, cyclosporin A	292
Side-chain modifications	Modifying side-chains of amino acids involved in protease recognition	Stabilizes peptide by preventing enzyme cleavage while preserving peptide activity	Prosaptide peptide derivatives of prosaposine (TX14)	293
Chirality changes	Replacing l-amino acids with d*-*amino acids to enhance resistance to proteolytic degradation and reduce immunogenicity	Improves protease resistance and reduces immunogenicity. Enhances the stability and activity of the peptide	d*-*Amino acid analogues of neurotrophic peptides, as used in peptide drug development	294

#### 3.4.1 N- and C-terminal modifications

In serum or plasma, small peptides are primarily degraded by exopeptidases, with all peptides possessing free *N*- and C-termini being particularly susceptible to rapid enzymatic degradation, often within min in physiological conditions. However, certain endogenous hormones and neuropeptides exhibit natural end-protection, conferring enhanced stability. For instance, thyrotropin-releasing hormone (TRH) is stabilized by an *N-*terminal pyroglutamyl residue and a C-terminal proline-amide moiety, while *α*-melanocyte-stimulating hormone (*α*-MSH) is protected by *N-*terminal acetylation and C-terminal amidation, rendering it susceptible only to endopeptidase-mediated degradation *in vivo*^295^. The acetylation process is catalyzed by acetyl-coenzyme A, whereas peptidylglycine-amidation monooxygenase facilitates C-terminal amidation^296^. These natural modification processes are important in preserving the biological activity and stability of many neuropeptides, and chemical modification methods mimic their effects^297^. End-protection strategies have been extensively employed in peptide drug development to enhance enzymatic stability. For example, N-terminal acetylation of a somatostatin analogue extended its *in vivo* half-life from 3 min to over 400 min^298^. While effective in delaying degradation, end-protection rarely confers complete resistance to enzymatic metabolism. Instead, it primarily slows peptide catabolism, as degradation still can continue *via* specific endopeptidases.

Beyond enzymatic stability, N-terminal modifications such as acetylation and lipidation can modulate peptide lipophilicity, which may theoretically enhance membrane permeability, particularly across physiological barriers such as the intestinal epithelium and the blood‒brain barrier (BBB). However, permeability enhancement is not universally observed. For instance, acetylated enkephalin analogues demonstrated reduced permeability across an *in vitro* model of the BBB using bovine brain endothelial cells^299^. It was proposed that the acetylated peptide exhibited increased cellular uptake but remained trapped intracellularly rather than traversing the endothelial monolayer^299^.

The lipidation of polypeptides with fatty acids to create *N-*linked lipopeptides can be a complex and labor-intensive procedure, frequently necessitating the protection of other reactive functional groups present on the amino acid side chains. Although lipidation is usually performed on N- or C-termini, lipidation can also be done on residues within a peptide chain. Recent advancements in chemical synthesis, particularly Cysteine Lipidation on a Peptide or Amino Acid (CLipPA) technology, have simplified this process^300^. CLipPA facilitates the direct lipidation of unprotected peptides that possess a free thiol group, resulting in the formation of *S-*lipidated lipopeptides. This method allows for the efficient and rapid synthesis of multiple lipopeptide analogs from a single thiolated polypeptide precursor^301^. Similar to N-linked lipidation, *S-*lipidation can enhance peptide-membrane interactions and intracellular retention, potentially improving cellular uptake while limiting transendothelial transport^302^. The impact of these modifications on *in vivo* BBB permeability remains to be elucidated^299^.

#### 3.4.2 Cyclization

Cyclization is a way to impose structural constraints that reduce conformational flexibility, thereby improving receptor binding affinity, selectivity, enzymatic resistance, bioavailability, and membrane permeability. This approach has been explored extensively in the design of orally administered cyclic peptides, including vasopressin and opioid analogues^303^^,^^304^.

Cyclic peptides can be classified as homodetic or heterodetic, depending on the nature of the linkage forming the ring structure. Homodetic cyclic peptides are defined by an intramolecular peptide bond between the N- and C-termini, whereas heterodetic cyclic peptides feature alternative bridging elements such as ether, disulfide, thioether, or ester (lactone) bonds. In addition, modern synthetic strategies, including all-carbon staples (*e.g.*, hydrocarbon stapling) and click chemistry-based linkages (*e.g.*, triazoles), are increasingly employed to generate heterodetic macrocycles^305^^,^^306^. Numerous chemical modifications have been explored and applied to modify linear peptides into cyclic structures. Among these, a widely used approach for synthesizing heterodetic cyclic peptides involves the formation of a disulfide bond between two cysteine residues, which effectively stabilizes the cyclic conformation^307^. However, in cases where cysteines are absent from the native peptide sequence, their incorporation may necessitate sequence modifications, potentially altering biological activity and receptor interactions^307^. Despite these challenges, cyclization remains a promising approach for optimizing peptide therapeutics by improving pharmacokinetic and pharmacodynamic properties and potentially removing N-terminal and C-terminal degradation sites. Advances in peptide engineering continue to refine cyclization strategies, expanding the potential of cyclic peptides for clinical applications.

#### 3.4.3 Methylation of amide nitrogen

Endopeptidases cleave peptide bonds, and as a result, *N-*methylation of the amide nitrogen within the peptide bond is a commonly utilized strategy to improve peptide stability. This modification enhances resistance to enzymatic degradation, particularly by endopeptidases, thereby prolonging the therapeutic efficacy of peptides^308^. This modification has been extensively investigated in the development of *N-*methylated enkephalin analogues, which retain their biological activity while demonstrating significantly improved metabolic stability^309^. Similarly, *N-*methylated derivatives of substance P, a neuropeptide involved in smooth muscle contraction and vasodilation, exhibit increased resistance to proteolytic enzymes without compromising potency^310^^,^^311^. A notable example of a naturally occurring *N-*methylated peptide is cyclosporin A, a cyclic undecapeptide with immunosuppressive properties. Cyclosporin A contains one unnatural amino acid in the form of 4-butenyl-4-methyl-threonine, and features N-methylation on seven of its eleven peptide bonds, contributing to its exceptional oral bioavailability and enzymatic stability^310^^,^^312^. However, *N-*methylation imposes structural constraints that can influence peptide conformation. Specifically, it disrupts hydrogen bonding interactions and promotes a *cis*-amide configuration, which may affect biological activity. For instance, reduced potency observed in *N-*methylated neurotensin analogues has been attributed to the loss of crucial intramolecular hydrogen bonds and conformational incompatibilities with the target receptor^313^. Therefore, while *N-*methylation is a powerful tool for enhancing peptide stability, its impact on structural dynamics and bioactivity must be carefully evaluated to ensure the retention of therapeutic efficacy. A rational design approach that balances stability and functional integrity is essential for optimizing peptide-based drug candidates.

#### 3.4.4 Side-chain modifications

A straightforward strategy to enhancing peptide and protein stability involves modifying the side chains of amino acids located at protease recognition sites^314^^,^^315^. Substituting these residues with natural or non-natural amino acids that possess chemically similar side chains can preserve the biological activity of the peptides and proteins while preventing enzymatic cleavage^314^^,^^315^. Altering the enzyme recognition section of peptides effectively enhances metabolic stability without significantly compromising the peptide's native structure and function. A well-documented example of this approach is the development of prosaptide peptide derivatives of prosaposin, a neurotrophic and neuroprotective agent. One such derivative, TX14, a tyrosine-sulfated prosaptide analog, demonstrated potent activity in the peripheral nervous system but exhibited limited efficacy in the central nervous system (CNS) due to rapid enzymatic degradation in the brain^316^.

#### 3.4.5 Chirality changes

Early pioneers in peptide and protein chemistry recognized the critical role of stereochemistry in dictating the structural and functional properties of these biomolecules. Their investigations revealed that stereochemical selectivity profoundly influences biological recognition, binding affinity, and functional specificity, laying the foundation for modern peptide-based drug design and protein engineering^316^, ^317^, ^318^, ^319^. The stereochemical specificity inherent in natural proteins, containing exclusively l*-*enantiomer amino acids, plays a critical role in molecular recognition, folding, and biological activity^316^, ^317^, ^318^, ^319^. This specificity is fundamental to the precise interactions between peptides and their receptors, enzymes, and other biomolecules. The strategic incorporation of d*-*amino acids into peptide sequences, either partially or completely replacing the natural l-amino acids, has gained recognition as an effective approach to improve peptide stability. d*-*Amino acid analogues are less susceptible to enzymatic degradation, as they are not readily recognized by the majority of proteolytic enzymes, thus extending their half-life in biological systems^320^. Moreover, d*-*amino acid-containing peptides have demonstrated significantly lower immunogenicity compared to their l-amino acid counterparts, which play a significant role in peptide and protein drug design and development. This reduction in immunogenicity not only improves the safety profile of these peptides but also enhances their potential for clinical applications, particularly in chronic treatments where long-term exposure to the peptide may otherwise elicit immune responses^316^, ^317^, ^318^, ^319^. Consequently, the incorporation of d*-*amino acids represents a valuable approach in designing peptides and proteins with improved pharmacokinetic properties, reduced immunogenicity, and enhanced therapeutic efficacy. Some examples and applications of chirality changes have been listed in^321^, ^322^, ^323^, ^324^ Table 8.

**Table 8:** Examples of peptide drug modifications by chirality changes and their benefits^321^, ^322^, ^323^, ^324^.

Drug	Modification	Benefit	Ref.
Desmopressin	d*-*Arg at position 8; deamination at position 1	Enhanced *T*_50_ 10 times	321
Difelikefalin	d*-*Phe at positions 1 & 2; D-Leu at position 3	High *κ*-opioid activity, protease-stable	322
Etelcalcetide	C*-*terminal d-amino acid backbone	Low immunogenicity	323
Degarelix	Multiple d*-*amino acids + hydrophobic substitutions	Enhanced GnRH receptor binding; increased plasma *T*_50_	324

### 3.5 Formulation strategy of nanoparticulate drug delivery systems

**Figure 5:**
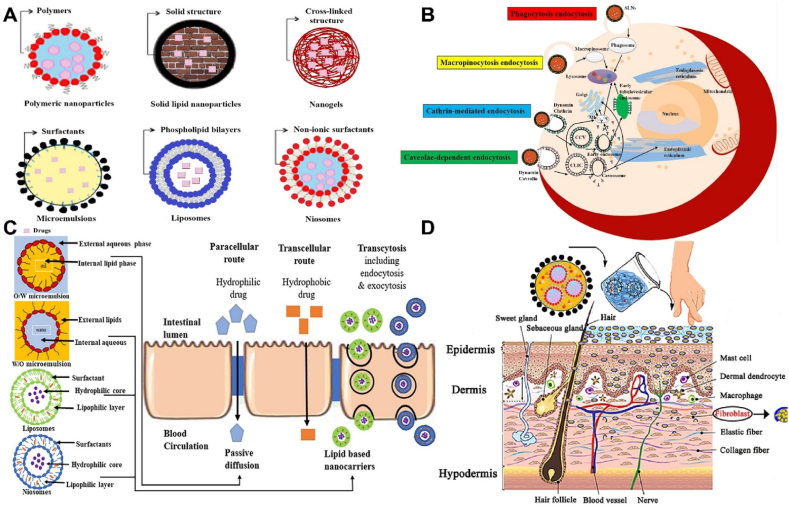
(A) Schematic representations of various nanoparticulate drug delivery carriers for peptide therapeutics, including polymeric nanoparticles, solid lipid nanoparticles (SLN), nanogels, microemulsions, liposomes, and niosomes. (B) Illustration of the different endocytic pathways involved in nanocarrier uptake. (C) Mechanisms by which nanocarriers traverse intestinal barriers for oral drug delivery. (D) Mechanisms by which nanocarriers penetrate skin barriers for topical and transdermal drug delivery.

Drug delivery *via* various administration routes could be developed by tailoring the particle size and physicochemical properties of carrier systems. One of the most effective strategies for enhancing the stability and therapeutic efficacy of peptides and proteins is their incorporation into nanoparticulate drug delivery systems, which have been extensively investigated to address the inherent stability challenges of peptide-based therapeutics^325^^,^^326^. Nanoparticles, defined as multiphase carrier vesicles with a particle size below 1000 nm, can encapsulate a broad range of bioactive compounds, thereby improving their stability, bioavailability, and therapeutic performance^326^^,^^327^. Compared to conventional drug delivery systems, such as microspheres and microparticles, nanoparticulate formulations offer several advantages: (1) high stability and drug-loading capacity, enabling the encapsulation of both hydrophilic and hydrophobic compounds while being compatible with multiple administration routes, including oral, injectable, and inhalation delivery^328^; (2) the capacity to deliver drugs in a modified and controlled release pattern, thereby prolonging therapeutic effects; and (3) enhanced resistance to enzymatic peptidolysis and proteolysis in the GIT and bloodstream, improving cellular uptake *via* endocytosis, phagocytosis, and micropinocytosis^329^. These properties collectively enhance drug bioavailability while reducing dosing frequency^328^. Common nanoparticulate delivery systems encompass liposomes, microemulsions, nanoparticles, nanogels, and niosomes, as depicted in Fig. 5A^330^. As presented in Table 9 333, 334, 335, 336, 337, 338, 339, 340, 341, 342, 343, 344, this overview summarizes advanced delivery systems developed for peptide and protein therapeutics, highlighting representative examples of both marketed drugs and clinical-stage candidates. These delivery platforms are designed to enhance stability, bioavailability, and controlled release, while also offering protection against enzymatic degradation and physiological barriers, thereby improving the overall therapeutic performance of biologics^331^. The cellular uptake of nanocarriers is mediated by multiple endocytic pathways, including clathrin-dependent and caveolae-mediated endocytosis, facilitating efficient intracellular drug delivery, as depicted in Fig. 5B ^332^. The release kinetics of encapsulated peptides and proteins are influenced by factors such as lipid matrix composition, particle size, and preparation techniques, allowing for sustained and controlled drug release. Optimization of surfactant concentration and lipid structure can minimize burst release while enhancing drug retention. These characteristics position nanoparticulate formulations as a promising approach for improving therapeutic outcomes across diverse administration routes, including oral, topical, dermal, and transdermal delivery, as demonstrated in Fig. 5C and D.

**Table 9:** Overview of key delivery systems for peptide and protein therapeutics, highlighting examples of marketed drugs and clinical-stage candidates^333^, ^334^, ^335^, ^336^, ^337^, ^338^, ^339^, ^340^, ^341^, ^342^, ^343^, ^344^.

Delivery system	Marketed drugs	Clinical-stage candidates	Ref.
Microemulsion	Cyclosporine A, Saquinavir, Neoral®	OPBP-1, ORMD-0801	333,334
Liposome	Doxil®, Marqibo®, Oral-Lyn™	Semaglutide hybrid, oral EPO, Biphasix™, BLP25/Stimuvax®	335,336
Niosome	None (cosmetics only, *e*.*g*., Lancome)	TP5 niosomes, catechin niosomes, Diclofenac niosomal gel	337,341
Nanogel	Eligard®	pH-responsive nanogels, HA nanogels, cCHP Nanogel	338,342
Polymeric NPs	Sandostatin LAR®, Bydureon®, Afrezza®, Zoladex®, Lupron Depot®	Oral insulin (spore-based), RL-QN15 NPs	339,343
PEGylation	Pegasys®, Neulasta®, Doxil®, Cimzia® (certolizumab pegol), Yorvipath (palopegteriparatide), Palynziq® (pegvaliase)	PT302 (exenatide), PEGylated brain targets, NLY01 (PEGylated exenatide)	340,344

#### 3.5.1 Microemulsion

Microemulsions are clear, thermodynamically stable, and isotropic mixtures consisting of oil, water, surfactants, and co-surfactants, typically featuring droplet sizes below 100 μm^345^^,^^346^. They can be categorized into three primary classifications: oil-in-water (O/W), water-in-oil (W/O), and bicontinuous microemulsions. The choice of surfactants influences the phase properties of the microemulsion, which can significantly enhance drug solubilization and protect peptide and protein drugs from enzymatic degradation^345^. For example, the bioavailability of the water-soluble peptide SK&F-106760 (arginylglycylaspartic acid) increased more than tenfold when incorporated into a W/O microemulsion with a mean droplet size of 15 nm, compared to the bioavailability of the pure drug solution^347^. However, the application of microemulsions is constrained by their relatively low drug loading capacity and the inclusion of unnecessary excipients, which can exhibit toxic effects when administered at high doses.

**Figure 6:**
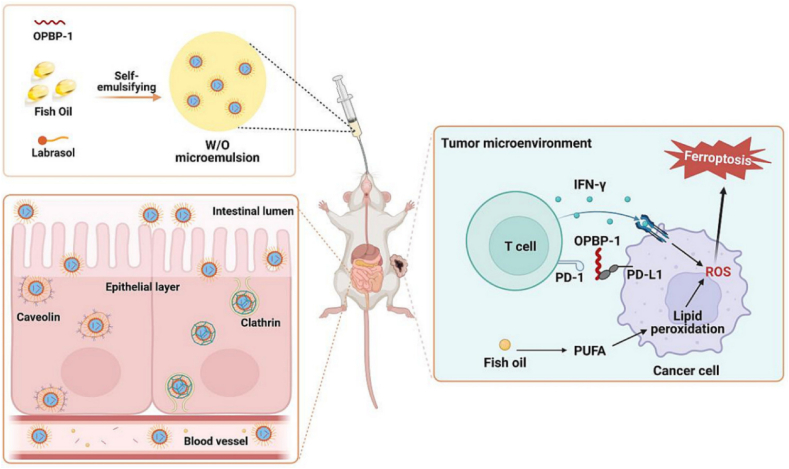
Schematic of a fish oil-based microemulsion for oral delivery of a PD-1/PD-L1 blocking peptide for cancer immunotherapy. Reprinted with the permission from Ref. 348. Copyright © 2023 Elsevier B.V.

A promising advancement in microemulsion-based peptide delivery is the development of a fish oil-based system for oral administration of the PD-1/PD-L1 blocking peptide OPBP-1, as shown in Fig. 6^348^. This formulation, prepared without the need for co-surfactants, exhibited favorable physicochemical properties, including a transparent, light-yellow appearance, good flowability, and a droplet size of 152 ± 0.73 nm. It demonstrated sustained drug release (56.45 ± 0.36% over 24 h) and remained stable under harsh intestinal conditions. *In vitro* studies confirmed enhanced peptide uptake and transport across Caco-2 cells, resulting in a 4.1-fold increase in oral bioavailability compared to the peptide solution. Mechanistic analysis identified clathrin- and caveolae-mediated endocytosis as the primary absorption pathways. *In vivo*, this system facilitated CD8^+^ T cell infiltration in tumors, increased IFN-*γ* secretion, and significantly suppressed murine colonic carcinoma (CT26) growth. Notably, fish oil-induced ferroptosis in tumor cells, demonstrating a synergistic effect with OPBP-1 in cancer immunotherapy. These findings highlight fish oil-based microemulsions as a promising, naturally derived platform for oral peptide delivery, leveraging their ability to enhance bioavailability while promoting reactive oxygen species (ROS)-mediated ferroptosis in tumors.

**Figure 7:**
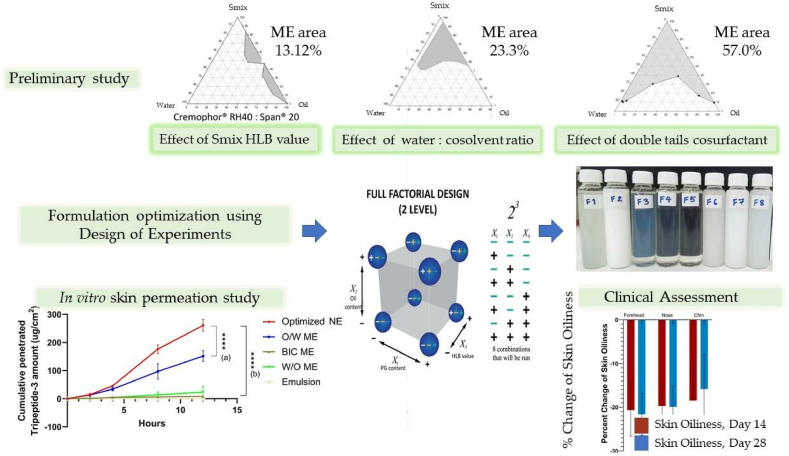
Schematic of microemulsions for topical delivery of tripeptide-3 for anti-sebum efficacy on facial skin. Reprinted with the permission from Ref. 333. Copyright © 2024 MDPI.

Another promising application of microemulsion-based peptide delivery is its use in skin treatments, particularly for facial oil control and skin hydration. A hydrophilic tripeptide-3 nanoemulsion was developed to enhance skin penetration and reduce sebum production, as illustrated in^333^ Fig. 7. The formulation process was optimized using water titration and pseudoternary phase diagrams, identifying key factors such as surfactant selection, hydrophilic-lipophilic balance (HLB), and co-solvent composition. A combination of Cremophor® RH40 and polyglycerol-3-diisostearate at an HLB of 13, along with a water-to-co-solvent (propylene glycol) ratio of 1:1, significantly reduced interfacial tension, facilitating stable nanoemulsion formation. Employing a low-energy emulsification method, the optimized formulation achieved a translucent oil-in-water nanoemulsion with a droplet size of 25.7 ± 1.20 nm, a narrow polydispersity index 0.237 ± 0.129, and high transmittance 70.6 ± 0.58%. *In vitro* skin permeation studies demonstrated superior penetration and retention of Tripeptide-3 compared to high-surfactant microemulsions and conventional emulsions. A 28-day clinical evaluation in healthy volunteers confirmed reduced sebum secretion and improved skin hydration without irritation. Together with the oral fish oil-based microemulsion for PD-1/PD-L1 blockade in cancer therapy, this study underscores the versatility of microemulsion systems in peptide delivery. These formulations improve both bioavailability and therapeutic effectiveness while also enabling targeted delivery for systemic and dermatological treatments.

#### 3.5.2 Liposomes

Liposomes are spherical vesicles, ranging in particle size from 1 to 2000 nm, consisting of one or more layers of phospholipids^349^. They offer several advantages for dermal and transdermal applications, including: 1) biocompatibility and biodegradability, 2) controlled drug release, 3) enhanced skin or topical deposition, 4) improved permeability, and 5) increased stability^350^, ^351^, ^352^. Numerous studies have shown that liposomes can improve the skin penetration of various actives for transdermal and topical applications, including tetracaine, corticosteroids, and ciclosporin^352^^,^^353^.

Studies have also been explored to increase the oral bioavailability of bioactives like insulin using liposomal formulations, with some studies reporting a decrease in plasma sugar levels following oral administration^354^, ^355^, ^356^. Similarly, other protein therapeutics, such as erythropoietin, calcitonin, and parathyroid hormone, have shown improved pharmacological effects when incorporated into liposomes for oral delivery^357^, ^358^, ^359^. However, liposomal formulations are not without their drawbacks. Issues such as hydrolysis, aggregation, fusion, and oxidation can lead to reduced stability and a reduced shelf life of liposomal products^360^. Additionally, the high cost of phospholipids contributes to the increased expense of liposomal formulations, limiting their widespread use^346^.

**Figure 8:**
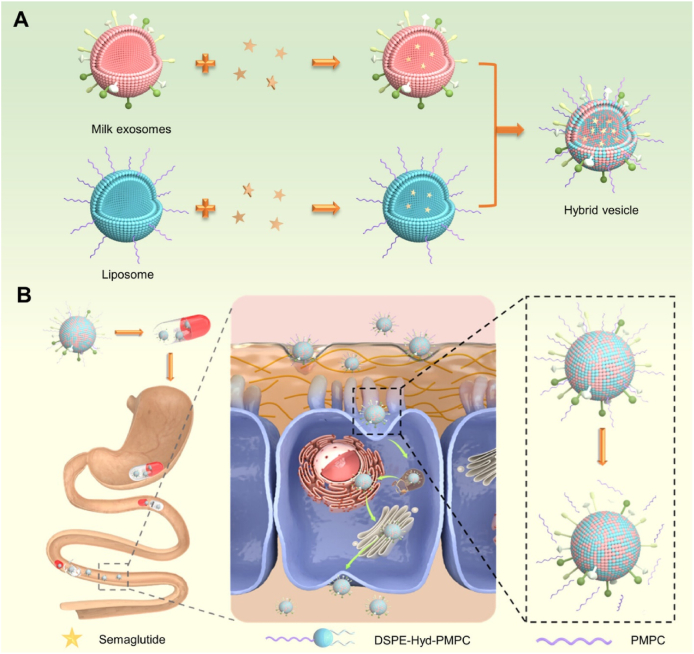
Schematic representative of exosome−liposome hybrid drug carrier for oral delivery of Semaglutide. (A) The preparation process and structural composition of the hybrid vesicle, and (B) the exosome−liposome vesicle effectively overcomes the GI tract and tissue barriers in the oral route. Reprinted with the permission from Ref. 335. Copyright © 2024 American Chemical Society.

A novel advancement in liposomal drug delivery is the development of hybrid vesicles that combine liposomes with exosomes, enhancing their stability and bioavailability for oral applications. One successful approach involves the fusion of functionalized liposomes with milk-derived exosomes to create a self-adaptive delivery system, as illustrated in^335^ Fig. 8. This hybrid vesicle leverages a pH-sensitive hydrazone bond between zwitterionic polymers and phospholipids, enabling surface property transformation in response to the pH microenvironment of the jejunum. In the intestinal lumen, the hydrophilic and neutrally charged surface of the hybrid vesicle facilitates mucus penetration while preserving membrane proteins. Upon reaching jejunal epithelial cells, the vesicle undergoes a charge shift, improving cellular uptake and intracellular transport. This adaptive mechanism enhances oral bioavailability to 8.7% and significantly improves therapeutic efficacy. This advancement builds on prior research demonstrating the potential of liposomes for oral protein and peptide drug delivery. Liposomal formulations have been explored to improve the bioavailability of insulin, erythropoietin, calcitonin, and parathyroid hormone, showing enhanced pharmacological effects. However, conventional liposomes face limitations such as hydrolysis, aggregation, and high production costs. The integration of exosomes addresses these challenges by improving liposomal stability and transport efficiency, representing a promising strategy for next-generation oral delivery systems.

**Figure 9:**
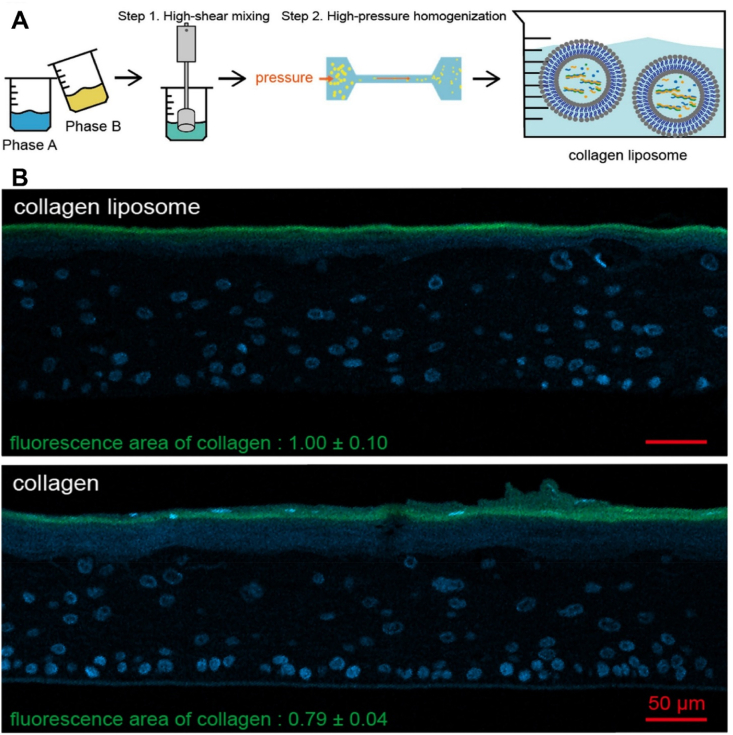
Schematic of liposome-assisted penetration and antiaging effects of collagen. Reprinted with the permission from Ref. 361. Copyright © 2023 Wiley Periodicals LLC.

Another promising skin application of liposomal technology is its role in enhancing collagen delivery for anti-aging effects, as illustrated in Fig. 9^361^. Collagen-encapsulated liposomes, prepared *via* high-pressure homogenization, demonstrated superior skin penetration and retention compared to native collagen. Significantly, these vehicles prolonged collagen retention in the artificial membranes by a factor of two, even after several wash cycles. Additionally, real-time PCR analysis demonstrated that 3D skin models in the presence of collagen-encapsulated liposomes showed increased expression of keratin, involucrin, and collagen, even at the post-exposure to ethanol. This suggests that liposomal vehicles not only enhance collagen stability and absorption but also promote skin regeneration and barrier function, making them effective vehicles for anti-aging formulations. This application aligns with advancements in liposomal delivery systems, including their integration with exosomes to improve oral bioavailability and stability. While conventional liposomes have been explored for oral protein delivery, challenges such as low stability and high production costs remain. The ability of liposomes to facilitate transdermal and oral delivery underscores their versatility in biomedical applications, from enhancing skin hydration and elasticity to improving systemic bioavailability of therapeutic peptides and proteins.

#### 3.5.3 Niosomes

Niosomes are non-ionic surfactant-based vesicular systems, structurally analogous to liposomes, but composed primarily of non-ionic surfactants and cholesterol. They form bilayered vesicles capable of encapsulating both hydrophilic and lipophilic drugs, offering improved stability, biocompatibility, and drug delivery potential. This unique niosomal structure offers several advantages: 1) niosomes are easier and more cost-effective to produce, 2) they enhance drug loading capacity, provide higher solubilization and encapsulation efficacy, particularly for the transdermal and topical delivery of peptide and protein drugs^362^. For instance, Manosroi et al.^363^ showed improved delivery of human tyrosinase plasmid using cationic niosomes in an abdominal rat skin model, observing a six-fold enhancement in drug permeability compared to a pure drug solution. Nevertheless, the incorporation of non-ionic surfactants in niosomes can reduce their physicochemical stability^364^. These surfactants may cause particle aggregation, therefore limiting the overall niosomal stability^362^. Additionally, charged nanocarriers may exhibit toxicity to human cells, emphasizing the need for careful consideration of safety when developing niosomal products^365^.

**Figure 10:**
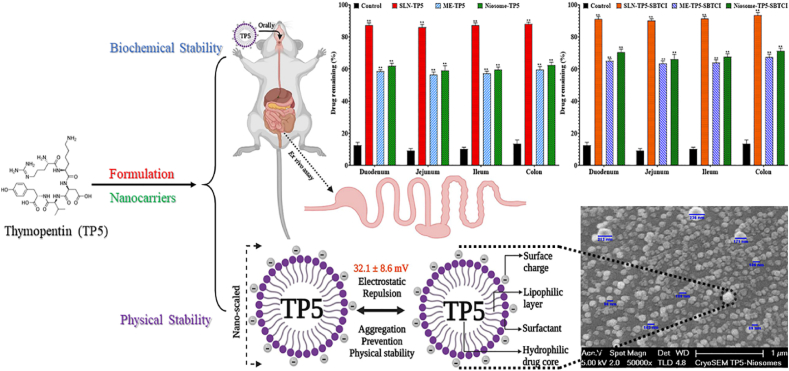
Schematic of lipid-based niosomes of TP5 for *ex vivo* studies towards oral application. Reprinted with the permission from Ref. 366. Copyright © 2022 Taylor & Francis Group.

A promising application of niosomes in oral drug delivery involves thymopentin (TP5), an immunomodulatory peptide prone to rapid degradation in the digestive system. Niosomal nanocarriers were fabricated using the thin film hydration method to enhance TP5 stability and prevent intestinal degradation. As shown in Fig. 10, TP5-niosomes exhibited superior protection against enzymatic degradation in *ex vivo* intestinal luminal contents and mucosal homogenates for up to 6 h, compared to the pure drug solution^366^. These findings suggest that lipid-based niosomes can effectively reduce peptide degradation, potentially improving the oral bioavailability of TP5.

**Figure 11:**
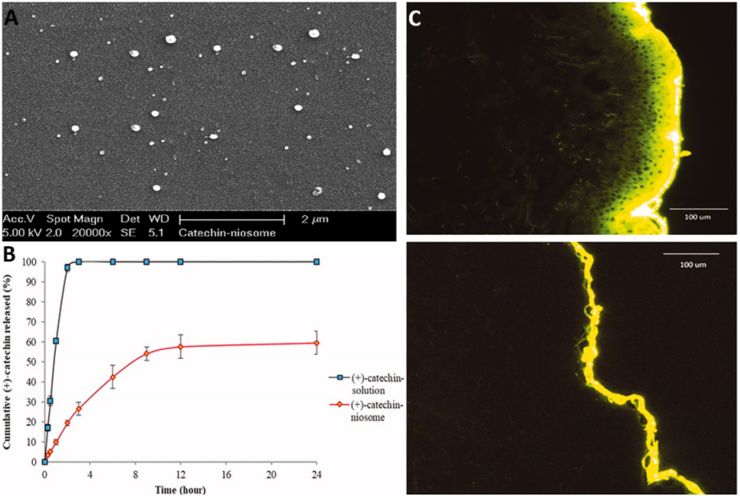
Non-ionic niosomes of catechin on skin application. (A) The morphology of catechin-loaded niosomes under SEM; (B) Drug release profiles of niosomes of catechin and its solution in 24 h; and (C) Fluorescence microscopic images of a cross-section of human skin treated with niosomes and ethanol solution control (15% ethanol in water). Reprinted with the permission from Ref. 367. Copyright © 2020 Taylor & Francis Group.

In dermatological applications, niosomes have been explored for the topical delivery of (+)-catechin, a potent antioxidant. Catechin-loaded niosomes, developed *via* film hydration, exhibited spherical nanoscale size (204 nm), high drug entrapment efficiency (49%), and a sustained drug release profile up to 24 h, as shown in Fig. 11A and B^367^. *Ex vivo* studies demonstrated significantly improved drug deposition and penetration in the viable layers of human skin (*P* < 0.05), and indicated enhanced cellular protection through an endocytosis-mediated uptake process, as shown in Fig. 11C ^367^. These findings underscore the potential of niosomes as effective topical carriers for antioxidant and peptide delivery in skincare.

#### 3.5.4 Nanogels

Nanogels, or polymeric hydrogels, are networks at the nanometer scale (typically under 1000 nm) made up of swollen structures formed from amphiphilic or hydrophilic polyionic polymers, which may be derived from artificial synthesis or natural extraction^368^^,^^369^. Nanogels have emerged as an advancing and promising drug carrier because of their distinct characteristics and versatile applications. The promising properties of nanogels involve customizable physicochemical structures, the ability to deform and adapt within the nano-range, a high surface area for multivalent conjugations, and a significant water content. Furthermore, nanogels are made from biocompatible materials, offering high drug loading and enhanced stability. They enhance the efficacy of targeted delivery to specific cells and compartments, exhibit desirable immunomodulatory effects, and demonstrate sensitivity to environmental changes^370^, ^371^, ^372^. These characteristics make nanogels a compelling option for advanced drug delivery systems. As a nanocarrier, it can be directed to specific sites following injection into bodily fluids, with the versatility to adjust its physicochemical properties to address key biological barriers, such as evading the reticuloendothelial system, reducing renal clearance *via* the glomeruli, and minimizing nonspecific accumulation in various organs *in vivo*. Nanogels have been mainly designed to improve drug stability through several approaches, including: physical self-assembly of interactive polymers, polymerization of monomers in homogeneous or micro/nanoscale heterogeneous systems, cross-linking of pre-synthesized polymers, and template-assisted nanofabrication techniques^373^. A variety of natural biodegradable polymers are commonly used in nanogel development, such as heparin, hyaluronic acid, dextran, dextrin, poly-l-lysine, poly(*γ*-glutamic acid) (*γ*-PGA), pullulan, mannan, chitosan, and alginate. Additionally, synthetic biocompatible and biodegradable polymers like poly(*ε*-caprolactone) (PCL), poly(methyl methacrylate) (PMMA), poly(glycolic acid) (PGA), poly(d,l-lactic acid) (PLA), and poly(d,l-lactic-*co*-glycolic acid) (PLGA) are also frequently used. Importantly, many of these polymers have been approved by the US Food and Drug Administration (FDA) for human use, making them suitable candidates for the development of nanoparticulate delivery systems^374^.

**Figure 12:**
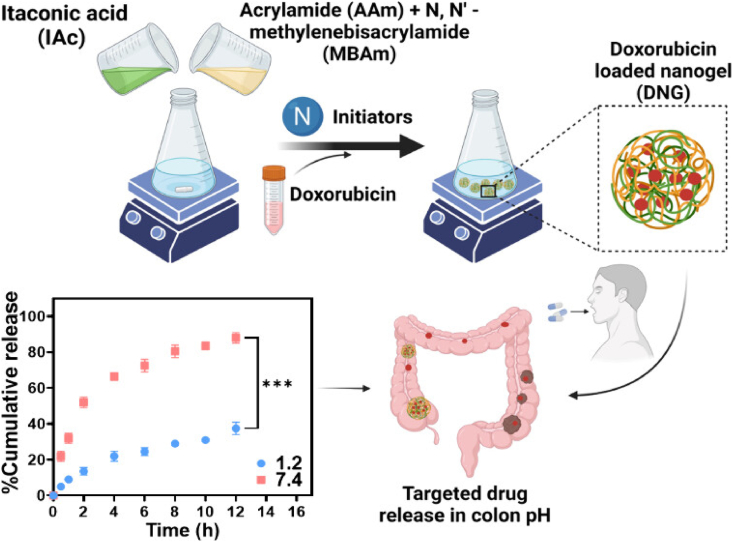
Schematic of pH-responsive swellable nanogel for colon-targeted oral drug delivery. Reprinted with the permission from Ref. 339. Copyright © 2024 American Chemical Society.

A promising example of nanogels in oral application is the development of pH-responsive, reactive oxygen species (ROS) scavenging, and highly swellable nanogels as shown in Fig. 12^342^. The nanogels are synthesized by acrylamide monomers with itaconic acid at a molar ratio of 4:1 through a free radical polymerization approach. The resulting spherical nanogels, measuring 180 ± 20 nm in size, are verified by field emission scanning electron microscopy. Dynamic light scattering analysis indicates a hydrodynamic diameter of 270 ± 25 nm and a surface charge of −6.9 ± 2.3 mV. The presence of various functional groups on the nanogels is confirmed through Fourier-transform infrared spectroscopy, X-ray photoelectron spectroscopy, and nuclear magnetic resonance analysis. The nanogels demonstrate a significant swelling (10-fold) at colonic pH (pH 7.4) compared to gastric pH (pH 1.2), highlighting their pH-responsive behavior. Furthermore, the nanogels exhibit a fivefold increase in ROS scavenging activity compared to the control.

**Figure 13:**
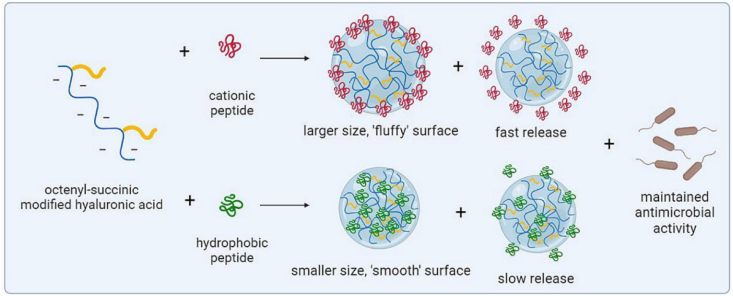
Schematic nanogel delivery systems for cationic peptides. Reprinted with the permission from Ref. 375. Copyright © 2024 Elsevier.

Hyaluronic acid-based nanogels have gained considerable attention in topical and transdermal delivery applications due to their self-assembling nature, biodegradability, and ease of preparation. A recent study explored the relationship between peptide structure and encapsulation efficiency within these nanogels, as illustrated in Fig. 13^375^. This study explored the effects of key peptide properties, such as charge and hydrophobicity, on their encapsulation within octenyl succinic anhydride-modified hyaluronic acid nanogels. The size and surface characteristics of the peptide-loaded nanogels, assembled using microfluidic techniques, were assessed through small-angle neutron scattering, laser Doppler electrophoresis, and dynamic light scattering. Moreover, this study explored changes in the protein's secondary structure following encapsulation, its release profile, and *in vitro* antimicrobial efficacy. The findings revealed that peptides with higher hydrophobicity demonstrated stronger interactions with the nanogel, preferentially localizing internally rather than at the surface. This interaction resulted in nanogels with smooth surfaces, a more spherical morphology, and prolonged release profiles. Conversely, cationic and hydrophilic peptides were primarily located at the surface of the nanogel, leading to looser structures and a faster, more complete release in biorelevant media. These resulting data underscore that the performance of nanogel delivery systems is influenced by the specific properties of the therapeutic peptides, highlighting the importance of peptide characteristics in tailoring nanogel formulations for targeted applications.

#### 3.5.5 Nanoparticles

Nanoparticles are defined as colloidal particles with sizes in the range below 1000 nm. As for transdermal delivery, the optimal particle size typically ranges below 400 nm. Nanoparticles can adopt a core‒shell structure, referred to as nanocapsules, or a continuous monolithic structure, known as nanospheres^376^^,^^377^. These nanoparticles offer significant advantages in improving the bioavailability of encapsulated therapeutic peptides and proteins. Research has demonstrated that encapsulating drugs in polymeric nanoparticles made from materials such as poly(lactic acid) (PLA)^378^, poly lactic-*co*-glycolic acid (PLGA)^379^, gelatin^380^ or chitosan^381^ enhances their stability and bioavailability. Particle size is a crucial factor influencing drug absorption, with smaller nanoparticles generally demonstrating superior absorption. Studies have shown that particles larger than 500 nm face significant challenges in being absorbed through the human skin^382^. Due to their small size, nanoparticles can also exhibit bioadhesion to the gastrointestinal tract wall, lodging in the intervillar spaces, which increases their residence time and enhances bioavailability^383^^,^^384^. Several studies have reported promising results from incorporating therapeutic peptides and proteins into solid lipid nanoparticles, such as thymopentin, streptacidin, and insulin^383^^,^^385^. However, polymeric nanoparticles may exhibit toxic side effects, making it essential to focus on developing nanoparticles with non-toxic characteristics and high stability for safer and more effective formulations.

**Figure 14:**
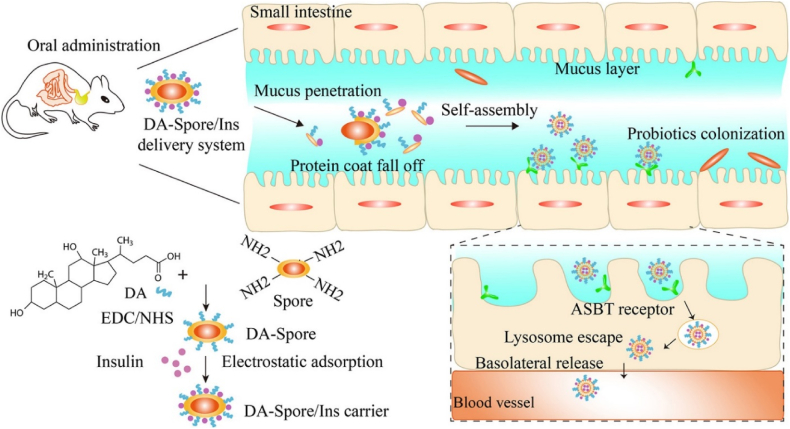
Schematic representation of a *Heyndrickxia coagulans* spore-based nanoparticle system designed for enhanced oral insulin delivery and hypoglycemic treatment. Reprinted with the permission from Ref. 386. Copyright © 2025 Elsevier.

One novel application for oral peptide and protein administration is motivated by the innate physicochemical characteristics of spores. This approach involves the use of deoxycholic acid-modified *Heyndrickxia coagulans* spores, which are loaded with insulin (DA-Spore/Ins). This system aims to overcome absorption barriers and enhance the oral delivery of insulin. As illustrated in Fig. 14, the DA-Spore/Ins formulation offers superior stability and facilitates effective mucus permeation *via* spore germination in the digestive microenvironment^386^. The system self-assembles into nanoparticles (NPs) by disintegrating the DA-covalently amphipathic protein coat and the hydrophilic protein/peptide drug, enabling efficient transport across epithelial cells *via* the bile acid pathway. *In vivo* studies showed that the DA-Spore/Ins system achieved a 15.1% oral relative bioavailability and superior hypoglycemic effects in type I diabetic rats, with good biocompatibility. These findings suggest that the biological properties of *Heyndrickxia coagulans* spore-based nanogenerators hold promise for oral insulin and other protein drug therapies. This approach is linked to the broader application of nanoparticles in oral delivery systems. Nanoparticles, ranging from 1 to 1000 nm in size, offer improved bioavailability for encapsulated peptides and proteins. Nanocapsules (with core‒shell structures) and nanospheres (with continuous monolithic structures) have been optimized to improve drug absorption. Particle size plays a crucial role in drug absorption, with smaller particles (200–400 nm) being optimal for transdermal administration. Nanoparticles can improve bioavailability by enhancing stability and facilitating absorption through bioadhesion to the GI tract wall, increasing residence time. However, attention must again be given to ensuring non-toxicity and stability in the formulation.

**Figure 15:**
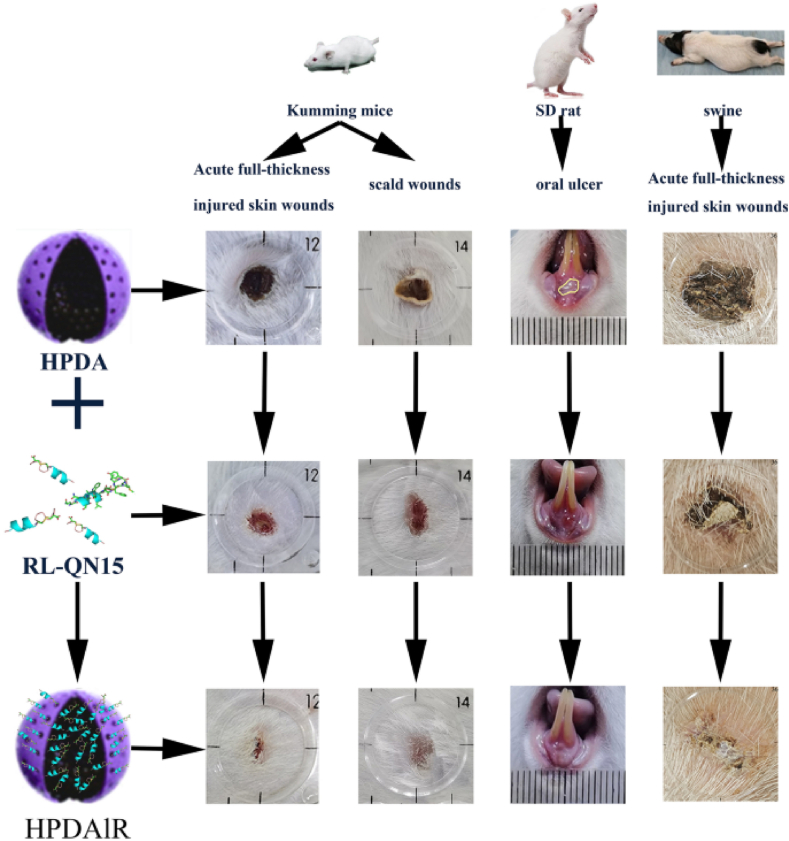
Schematic of hollow polydopamine nanoparticles loading with peptide RL-QN15 for skin wounds. Reprinted with the permission from Ref. 341. Copyright © 2021 Springer Nature.

Hollow polydopamine nanoparticles (HPDA) have shown promise for delivering the pro-healing peptide RL-QN15 to skin wounds. As shown in Fig. 15, HPDA nanoparticles were synthesized and loaded with RL-QN15 (HPDAlR), an amphibian-derived peptide with pro-healing properties^343^. The characterization, biodistribution, and clearance of both HPDA nanoparticles and HPDAlR were systematically examined. Additionally, the loading efficiency of RL-QN15 and its sustained-release profile from HPDAlR were also assessed. Both HPDA nanoparticles and HPDAlR exhibited non-toxicity towards keratinocytes, macrophages, and mice. Although HPDA nanoparticles did not show significant pro-healing effects, HPDAlR significantly improved the therapeutic efficacy of RL-QN15, accelerating wound healing and modulating cytokine release from macrophages. In animal models, HPDAlR showed a 50-fold increase in regenerative potency for mouse skin wounds and a 10-fold increase in oral ulcers in rats compared to RL-QN15 alone. Additionally, HPDAlR accelerated healing in skin scalds in mice and full-thickness skin wounds in swine. These findings suggest that HPDAlR holds significant potential for skin wound healing therapies, highlighting the growing role of nanoparticles in enhancing drug stability, controlled release, and tissue regeneration.

#### 3.5.6 PEGylated nanocarriers

**Figure 16:**
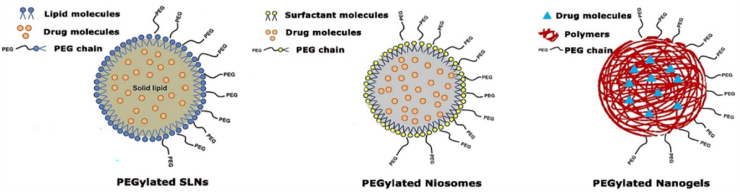
The proposed structures of PEGylated SLNs, niosomes, and nanogels.

PEGylation is chosen as the preferred additive as it is one of the most effective and common modifications among the nanoparticulate drug delivery systems^387^. PEGylation refers to the chemical modification of small molecules and therapeutic bioactives by attaching polyethylene glycol (PEG) molecules of varying lengths to improve their pharmaceutical properties^388^. PEGs are synthetic, highly water-soluble, and inert polymers that come in a wide array of molecular weights. These polymers are commonly found in consumer healthcare products, including laxatives, toothpaste, and shampoos, and are particularly prominent in biopharmaceutical applications^389^. The process of PEGylation offers several advantages, including prolonged circulation time of proteins or nucleic acids, enhanced water solubility of therapeutics, protection against biological inactivation (*e.g.*, peptidolysis and proteolysis), and a reduction in immunogenicity^390^. The primary benefit of PEGylation is its ability to extend the half-life of therapeutic agents, thus improving drug stability *in vivo* and reducing renal clearance of the drug^391^. PEGylating a drug delivery system improves systemic circulation time and decreases immunogenicity to obtain higher stability. The impact of PEG coatings on the surface of systemically administered nanoparticulate drug delivery formulations has, and continues to be, widely studied^392^. Some examples can be PEGylated SLNs, PEGylated Niosomes, and PEGylated nanogels, whose structures are shown in Fig. 16^393^. PEGylation of a nanoparticulate drug delivery system not only enhances the bioactives' *in vivo* stability but also improves the biocompatibility and therapeutic effect^394^.

**Figure 17:**
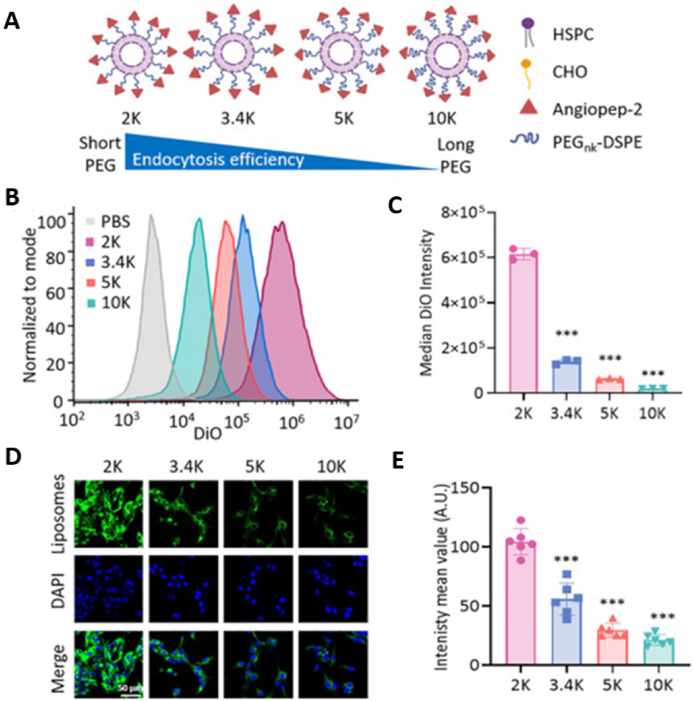
Schematic representation of PEGylated liposomes. (A) Composition of liposomes with varying PEG chain lengths; (B) Cellular uptake of U87MG-Luc cells incubated with PEGylated liposomes for 6 h; (C) Median DiO intensity of cells incubated with different PEGylated liposomes, as measured by flow cytometry. Data are presented as mean ± SD, *n* = 3; (D) Confocal laser scanning microscopy images of U87MG-Luc cells following 6 h of incubation with various PEGylated liposomes; (E) DiO fluorescence intensity in U87MG-Luc cells measured by Zen, mean ± SD, *n* = 6; Reprinted with the permission from Ref. 343. Copyright © 2024 Elsevier.

One of the PEGylated nanocarrier applications is PEGylated liposomes. In the study from Du et al.^344^, the influence of PEG chain lengths (2, 3.4, 5, and 10 kDa) on BBB penetration and brain targeting using Angiopep-2 peptide-decorated liposomes was explored, as shown in Fig. 17. The results revealed that shorter PEG chains facilitated more efficient *in vitro* cell uptake through endocytosis, while longer PEG chains enhanced BBB penetration *via* transcytosis. *In vivo*, liposomes with longer PEG chains showed superior brain accumulation in both normal and glioblastoma (GBM)-bearing mice due to prolonged circulation and improved BBB penetration. These findings highlight the critical role of PEG chain length in designing nanocarriers for targeted delivery, particularly for brain diseases. This study illustrates the potential of PEGylated nanocarriers for peptide delivery, offering key insights into the design of nanocarriers for enhanced bioavailability and targeted treatment. PEGylation, which involves attaching polyethylene glycol (PEG) molecules to the surface of nanoparticles, is widely used to improve the stability, solubility, and circulation time of therapeutic peptides. This modification not only enhances the biocompatibility and stability of the peptides but also facilitates their controlled release and efficient delivery to specific tissues, such as the brain. The varying lengths of PEG chains can significantly affect the properties of the nanocarriers, such as the release rate and bioadhesion, which is crucial for optimizing peptide delivery systems for clinical applications.

### 3.6 Optimization of administration route and anatomical site

In addition to chemical, physical, and nanotechnological strategies, the choice of administration route and anatomical site exerts a critical influence on the pharmacokinetic behavior, therapeutic efficacy, and patient acceptability of protein and peptide therapeutics^395^. Due to their large molecular size, structural fragility, and susceptibility to enzymatic degradation, these macromolecules often exhibit limited membrane permeability and poor oral bioavailability^396^. Consequently, delivery routes that circumvent first-pass hepatic metabolism and proteolytic barriers are pivotal for clinical success. The pulmonary route offers several pharmacological advantages owing to the lung's extensive absorptive surface area (∼100 m^2^), dense capillary network, and ultrathin alveolar epithelium (∼0.1–0.2 μm), which collectively facilitate rapid systemic absorption and avoidance of hepatic metabolism^397^. Approved products such as inhaled insulin (*e.g.*, Afrezza®) exemplify the feasibility of this approach^398^. Nevertheless, barriers such as mucociliary clearance, alveolar macrophage uptake, and enzymatic degradation in the epithelial lining fluid pose significant challenges. Moreover, efficient alveolar deposition necessitates particles with aerodynamic diameters between 0.5 and 3 μm, as larger particles are retained in the upper airways while smaller ones are exhaled^399^. Inhaled protein formulations also require stabilization against shear-induced aggregation during aerosolization and pH-sensitive degradation. Similarly, the nasal route has garnered attention due to its high vascularity and approximately 150 cm^2^ of absorptive surface, enabling rapid systemic uptake and, uniquely, direct nose-to-brain transport *via* the olfactory and trigeminal neural pathways^400^. This allows for the non-invasive delivery of neuropeptides and protein-based CNS therapies. However, short residence time (∼15–30 min), mucociliary clearance, and tight junctions limiting paracellular diffusion, in addition to enzymatic barriers such as aminopeptidases, compromise bioavailability^401^. Strategies to enhance mucosal permeation—such as mucoadhesive polymers, enzyme inhibitors, and surfactants like bile salts or chitosan—must be carefully optimized to balance absorption enhancement against risks of epithelial irritation and long-term toxicity. The buccal and sublingual mucosae provide direct access to systemic circulation *via* the jugular and facial veins, bypassing hepatic first-pass metabolism and minimizing enzymatic degradation^402^. These tissues are more enzymatically stable than the gastrointestinal tract, offering a relatively favorable milieu for peptide absorption. However, the stratified squamous epithelium (∼500–800 μm) presents a significant diffusion barrier, and the lack of active transport mechanisms restricts uptake primarily to low-molecular-weight, lipophilic peptides^403^. Advanced delivery strategies under development include mucoadhesive films, permeation enhancers, and electro-driven systems (*e.g.*, iontophoresis), although few have progressed beyond preclinical evaluation due to limited permeability and difficulty sustaining therapeutic levels. SC injection remains the most widely adopted route for peptide and protein drugs (*e.g.*, insulin analogues, GLP-1 receptor agonists) due to its relatively convenient administration and consistent systemic exposure^404^. However, SC absorption is influenced by local blood flow, enzymatic activity, and lymphatic drainage, resulting in inter- and intra-patient variability. By contrast, intradermal delivery, particularly *via* microneedle arrays, provides access to the richly vascularized dermis, enabling rapid onset, dose sparing, and improved patient adherence. Microneedle-mediated delivery also allows for targeted immune activation, making it a compelling platform for peptide vaccines and long-acting depot formulations^405^. Finally, site-specific routes, such as intra-articular, intravitreal, or intrathecal administration, are employed for localized therapeutic effects, especially in inflammatory, ophthalmic, or central nervous system disorders^406^. These specialized approaches offer high local concentrations while reducing systemic exposure, but necessitate highly controlled formulations with stringent sterility and stability requirements to mitigate risks of immunogenicity and toxicity. In overview, tailoring the delivery route and anatomical target site, in conjunction with formulation design, represents a fundamental strategy to overcome the pharmacological and biopharmaceutical limitations of peptide and protein therapeutics. A route-specific approach that accounts for anatomical barriers, enzymatic environments, and patient-centric considerations is indispensable for advancing these therapies from bench to bedside.

## 4 Perspectives and limitations

Peptide- and protein-based therapeutics hold significant clinical promise due to their high specificity, potent bioactivity, and favorable safety profiles. The recently approved protein and peptide drugs are selected and listed in Table 10 418, 419, 420, 421, 422, 423, 424, 425, 426, 427, 428, 429, 430, 431, 432, 433, 434. Based on this, a growing number of peptide and protein therapeutics have gained FDA approval in recent years, showcasing diverse innovations in formulation and delivery, ranging from oral absorption enhancers (*e.g.*, Rybelsus® and Mycapssa®) to long-acting injectables (*e.g.*, Cabenuva® and Mounjaro®) and advanced biologics such as cell and gene therapies (*e.g.*, Skysona® and Lantidra™). These advances highlight the evolving landscape of biologic drug delivery, where physicochemical challenges such as enzymatic degradation, poor membrane permeability, and short half-lives are being addressed through tailored delivery technologies.

**Table 10:** Examples of recently FDA-approved peptide and protein drugs^418^, ^434^.

Drug	Indication	Route	Approval year	Type	Formulation/Innovation	Ref.
Rybelsus® (Semaglutide)	Type 2 diabetes	Oral	2019	Peptide	SNAC-mediated absorption enhancer (Novo Nordisk)	418
Vyleesi® (Bremelanotide)	Hypoactive sexual desire disorder	SC	2019	Peptide	Melanocortin receptor agonist	419
Evenity® (Romosozumab)	Osteoporosis	SC	2019	Protein	Anti-sclerostin monoclonal antibody	420
Lyumjev® (insulin lispro-aabc)	Diabetes	SC	2020	Protein	Ultra-rapid formulation with citrate/treprostinil	421
Imcivree® (Setmelanotide)	Genetic obesity syndromes	SC	2020	Peptide	MC4R agonist	422
Cabenuva® (Cabotegravir + Rilpivirine)	HIV-1	IM	2021	Protein	Monthly long-acting injectable	423
Nulibry® (Fosdenopterin)	MoCD type A	IV	2021	Peptide	Cyclic pyranopterin monophosphate analogue	424
Mounjaro® (Tirzepatide)	Type 2 diabetes	SC	2022	Peptide	Dual GIP/GLP-1 receptor agonist	425
Terlivaz® (Terlipressin)	Hepatorenal syndrome	IV	2022	Peptide	Vasopressin analogue	426
Skysona® (Elivaldogene autotemcel)	Cerebral adrenoleukodystrophy	IV	2022	Protein	Gene-modified autologous cell therapy	427
Zegalogue® (Dasiglucagon)	Severe hypoglycemia	SC	2021	Peptide	Glucagon analogue with enhanced stability	428
Bkemv® (Pegcetacoplan)	Paroxysmal nocturnal hemoglobinuria	SC	2023	Protein	C3 complement inhibitor	429
Lantidra™ (Donislecel)	Type 1 diabetes	IV	2023	Protein	Allogeneic pancreatic islets	430
Ozempic®	Type 2 diabetes	SC	2022	Peptide	Extended dosing of GLP-1 analogue	431
Zepbound™ (Tirzepatide)	Obesity	SC	2023	Peptide	Dual GIP/GLP-1 agonist for chronic weight control	432
Octreotide (Mycapssa®)	Acromegaly	Oral	2020	Peptide	Enteric-coated capsule with permeability enhancers	433
Oramed ORMD-0801 (insulin)	Type 2 diabetes	Oral	2025–	Protein	Protease inhibitors with enteric coating (in trials)	434

Despite this positive progress, the full therapeutic potential of peptide and protein drugs remains partially unrealized, largely due to the inherent limitations and practical complexities of current delivery enhancement strategies. For example, nanoparticle- or carrier-based systems often involve multistep and resource-intensive manufacturing processes, which are difficult to scale up while maintaining product consistency and reproducibility^407^. Moreover, formulation complexity introduces challenges in quality control, stability assurance, and cost–effectiveness, limiting their accessibility and commercial viability. The use of excipients such as surfactants, co-surfactants, and stabilizers, although necessary for enhancing drug stability and bioavailability, can generate intermediate degradation products or excipient–drug interactions that compromise therapeutic efficacy or introduce safety concerns, especially when administered chronically^408^. These potential risks require comprehensive safety and toxicity testing, which can delay development timelines and increase regulatory scrutiny^409^. Furthermore, interpatient variability in pharmacokinetics, coupled with the possibility of immune responses to delivery vehicles or protein aggregates, adds an additional layer of complexity to clinical translation. Immunogenicity risks are especially concerning for repeated administration of modified proteins or formulations involving novel materials. In some cases, enhancement strategies such as PEGylation, while extending half-life, may inadvertently alter receptor binding or lead to accelerated blood clearance phenomena after repeated dosing, undermining long-term efficacy^410^. Thus, while promising strategies have emerged, bridging the gap between laboratory research and clinical implementation continues to demand multidisciplinary innovation, robust translational frameworks, and regulatory harmonization. A systematic approach during formulation development could mitigate these challenges. Strategies such as ‘quality-by-design’ and ‘formulation-by-design’ provide a rational and scientific framework for optimizing formulations, thereby improving their clinical translation potential^411^^,^^412^.

Regulatory considerations are also crucial for the clinical success of peptide/protein-loaded nanoparticulate drug delivery systems. Safety and toxicity assessments are essential when seeking regulatory approval, as these formulations often involve complex systems containing multiple excipients, such as surfactants, co-surfactants, and stabilizers. These excipients must be selected from the Generally Recognized as Safe (GRAS) list maintained by global regulatory agencies, ensuring their established safety and toxicity profiles^413^^,^^414^. However, specific regulatory guidelines for nanocarrier-based therapeutics remain lacking, as agencies such as the FDA and the European Medicines Agency (EMA) have yet to establish comprehensive frameworks for their approval^415^. To address this gap, the USFDA has formed a Nano-Technology Interest Group to facilitate communication and educate regulatory staff on advancements in nanomedicine^416^^,^^417^.

Looking forward, emerging technological trends are expected to further reshape the field. Intelligent delivery systems incorporating stimuli-responsive materials, AI-driven formulation design, and bioinspired delivery platforms (*e.g.*, exosome-mimetic carriers) offer transformative potential for overcoming current barriers. Additionally, convergence with digital health tools, such as smart injectors and wearable biosensors, may enable real-time dosing feedback and personalized therapeutic regimens. The integration of synthetic biology with delivery science is also anticipated to produce next-generation biotherapeutics with programmable release and enhanced tissue targeting. By aligning regulatory frameworks and industrial scale-up with such innovations, the field is poised to move toward more precise, effective, and patient-centric delivery of peptide and protein therapeutics.

Overall, while peptide/protein-based therapeutics offer significant clinical potential, overcoming challenges related to delivery, regulatory approval, and large-scale production remains essential. Through systematic formulation development, regulatory alignment, and innovative strategies such as chemical modifications and advanced nanoparticulate systems, the next generation of peptide and protein therapeutics can achieve improved efficacy, stability, and patient compliance.

## 5 Conclusions

This article has offered an in-depth analysis of the key challenges associated with peptide drug delivery, including physical, enzymatic, and membrane-related barriers that limit their clinical effectiveness. By examining the complexities of peptide metabolism and the challenges inherent to various administration routes, such as oral, parenteral, topical, and transdermal, this paper highlights the necessity for innovative approaches to improve peptide/protein bioavailability and therapeutic efficacy. Understanding the interplay between therapeutic peptides/proteins and their barriers to delivery to their site of action is essential for the development of advanced drug delivery systems that offer high entrapment efficiency, multifunctionality, and cost-effectiveness. The integration of chemical modifications, tailored formulation strategies, and nanoparticulate delivery systems holds significant potential for improving peptide stability, bioavailability, and patient adherence. Recent advancements in nanotechnology-based carriers, such as liposomes, polymeric nanoparticles, and nanoemulsions, have demonstrated promising outcomes in protecting peptides from enzymatic degradation and facilitating targeted delivery. Furthermore, systematic approaches, including quality-by-design and formulation-by-design, provide rational frameworks for optimizing peptide and protein formulations and streamlining their clinical translation. This review serves as a valuable resource for researchers in peptide and protein therapeutics, offering critical insights into formulation strategies, emerging trends, and translational pathways for overcoming delivery challenges. With continued research and innovation, there is considerable potential to bridge existing gaps in peptide- and protein-based drug delivery, ultimately realizing their full clinical potential and improving therapeutic outcomes.

## Author contributions

Mengyang Liu and Jingyuan Wen designed the research. Mengyang Liu wrote the manuscript. Mengyang Liu, Darren Svirskis, Thomas Proft, Jacelyn Loh, Naibo Yin, Hao Li, Danhui Li, Yongzhi Zhou, Shuo Chen, Lizhuo Song, Guanyu Chen, Wei-Yue Lu, Zhiwen Zhang, Zhou Zhou, Lian Li, Yuan Huang, Craig Bunt, Guiju Sun, Paul W.R. Harris, Margaret A. Brimble, and Jingyuan Wen revised the manuscript. All of the authors have read and approved the final manuscript.

## Conflicts of interest

The authors have no conflicts of interest to declare.
